# Cycloaddition and functionalization reactions involving tropone

**DOI:** 10.1039/d5ra05481h

**Published:** 2025-09-19

**Authors:** Fatemeh Doraghi, Mohammad Hadi Edareh, Bagher Larijani, Mohammad Mahdavi

**Affiliations:** a Endocrinology and Metabolism Research Center, Endocrinology and Metabolism Clinical Sciences Institute, Tehran University of Medical Sciences Tehran Iran momahdavi@sina.tums.ac.ir; b School of Chemistry, College of Science, University of Tehran Tehran Iran

## Abstract

Tropones are valuable moieties in the synthesis of natural products and bioactive molecules. Tropones have also emerged as fascinating synthetic partners for various (4 + 2)-, (4 + 6)-, (6 + 3)-, (6 + 4)-, (6 + 6)-, (8 + 2)-, and (8 + 3)-cycloaddition reactions as well as functionalization reactions. This review highlights various cycloaddition and functionalization reactions involving tropone building blocks reported since 2014.

## Abstract 2

Tropones and its derivatives are highly valuable and readily available seven-membered ring compounds that undergo both cycloaddition and functionalization reactions, yielding diverse products. This review highlights these reactions.

## Graphical abstract

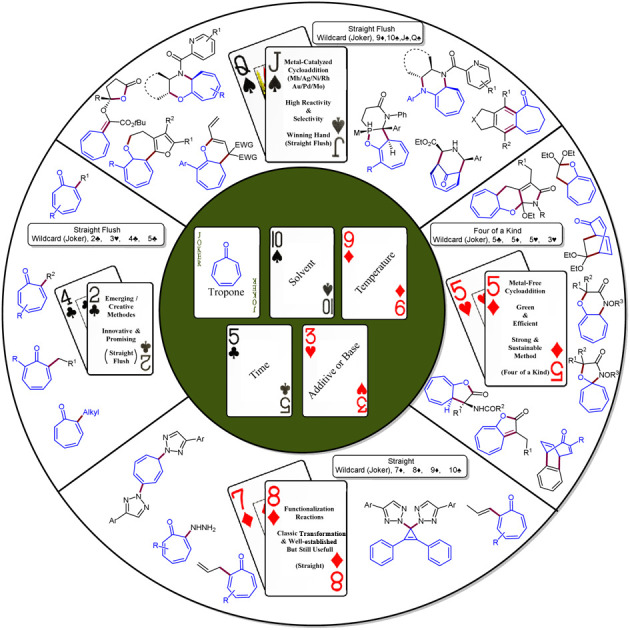

## 1. Introduction

**Fig. 1:**
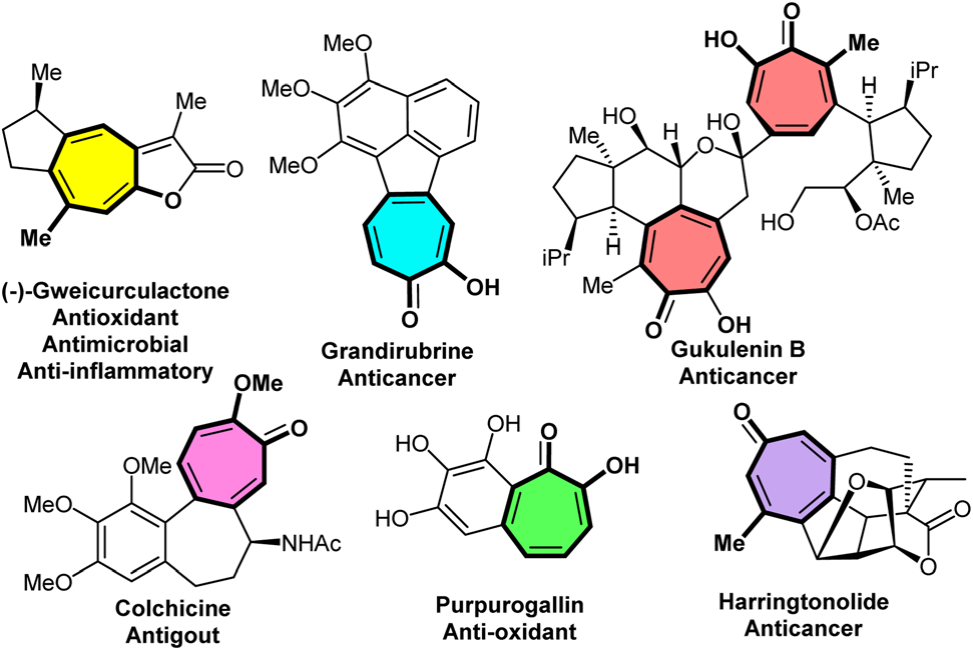
Representative pharmaceutical molecules containing a tropone motif.

Tropones are frequently present in numerous natural products,^1–4^ as well as agricultural^5^ and pharmaceutical^6,7^ molecules. (−)-Gweicurculactone (anti-oxidant, antimicrobial, anti-inflammatory),^8^ grandirubrine (anticancer),^9^ gukulenin B (anticancer),^10^ colchicine (antigout),^11^ purpurogallin (anti-oxidant),^12^ and harringtonolide (anticancer)^13^ are representative examples of densely polycyclic scaffolds that include the cycloheptatrienone core (Fig. 1). Tropones have also been widely used in organic synthesis and mechanistic studies to explore the chemistry and reactivity of these important synthons.^14–20^ They have been proved to be versatile 4π, 6π, or 8π synthons in (4 + 2)-,^21^ (4 + 6)-,^22^, (6 + 3)-,^23^ (6 + 4)-,^24^ (6 + 6)-,^25^ (8 + 2)-,^26^ and (8 + 3)-cycloadditions.^27^ In particular, higher-order cycloadditions exploit their distinctive extended π-conjugation, enabling efficient access to a wide range of bridged and fused cyclic skeletons of biological interest in the synthesis of medicinally important molecules.

**Fig. 2:**
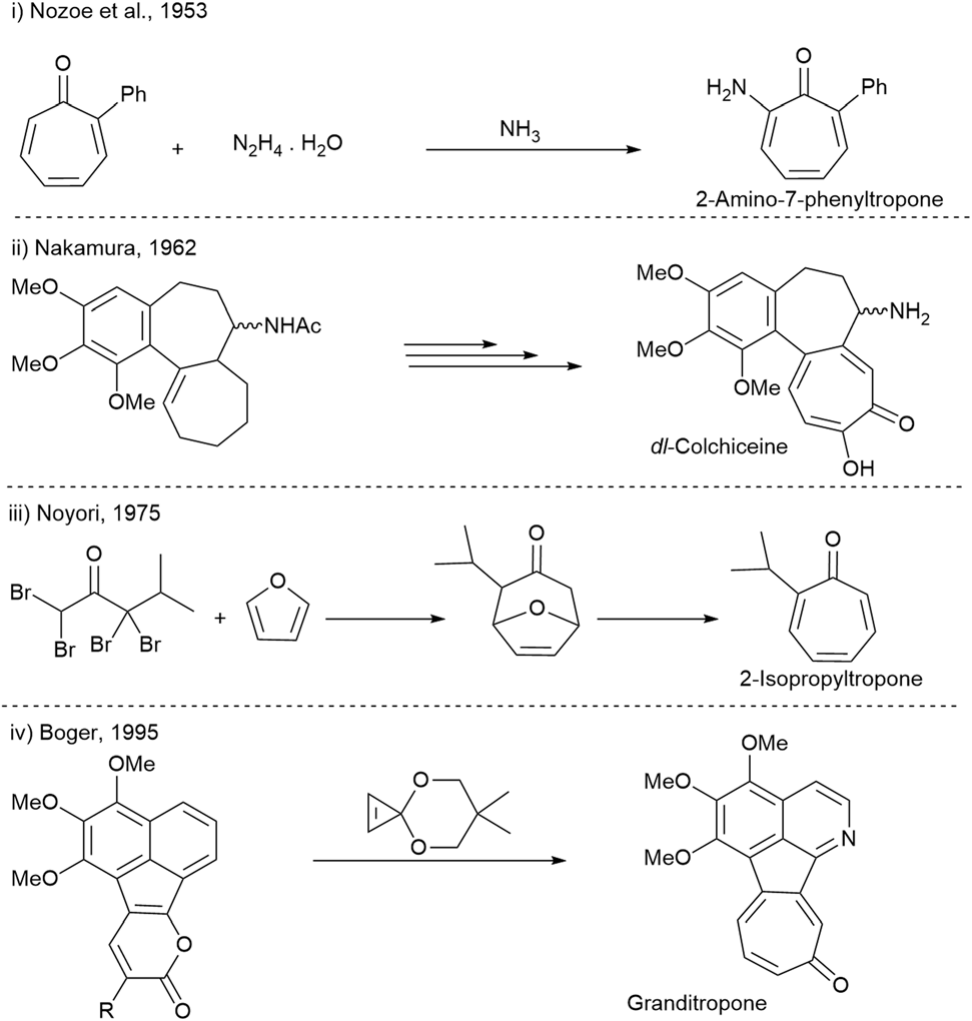
Primary synthetic methods in the synthesis of tropone derivatives: (i) Nozoe *et al.*, 1953 (ii) Nakamura, 1962 (iii) Noyori, 1975 (iv) Boger, 1995.

From a synthetic perspective, tropones are suitable substrates for a variety of functionalization reactions, retaining the cycloheptatrienone structure.^10^ In contrast to conventional aromatic compounds, little attention has been paid to tropones concerning direct C(sp^2^)–H bond functionalization, which constitutes a direct and atom-economical protocol towards structurally diverse substituted tropones. For instance, in 1953, Nozoe and coworkers reported the reaction of 2-phenyltropone with hydrazine hydrate, which led to the access of 2-amino-7-phenyltropone (Fig. 2(i)).^28^ Over the years, many research teams have reported the synthesis of tropone derivatives. For example, Nakamura presented total synthesis of colchiceine in 1962,^29^ and 1985,^30^ respectively (Fig. 2(ii) and (iv)). In 1975, Noyori reported a two-step reaction access to 2-isopropyltropone (Figure 2(iii)),^31^ and Boger, in 1995, developed a synthetic strategy for the assembly of granditropone (Fig. 2(v)).^32^

In recent years, the existing portfolio of tropone chemistry has focused on dearomative cycloaddition reactions, the *de novo* construction of seven-membered rings, and investigations into chemical tropone decorations *via* direct C–H bond functionalization.

Considering the notable importance of tropone in medicinal discovery and organic synthesis, numerous studies on the chemistry and reactivity of this valuable synthon have been conducted by several research groups. In this review, we highlight various cycloaddition and functionalization reactions involving tropone and its derivatives, which occur in the presence of metal catalysts such as Ag, Ni, Rh, Mg, Au, Pd, Mo, organocatalysts, bases, and under catalyst-free conditions.

## 2. Cycloaddition reactions involving tropone

### 2.1. Metal-catalyzed cycloaddition reactions involving tropone

#### 2.1.1. Mg-catalyzed cycloaddition reactions involving tropone

**Scheme 1:**
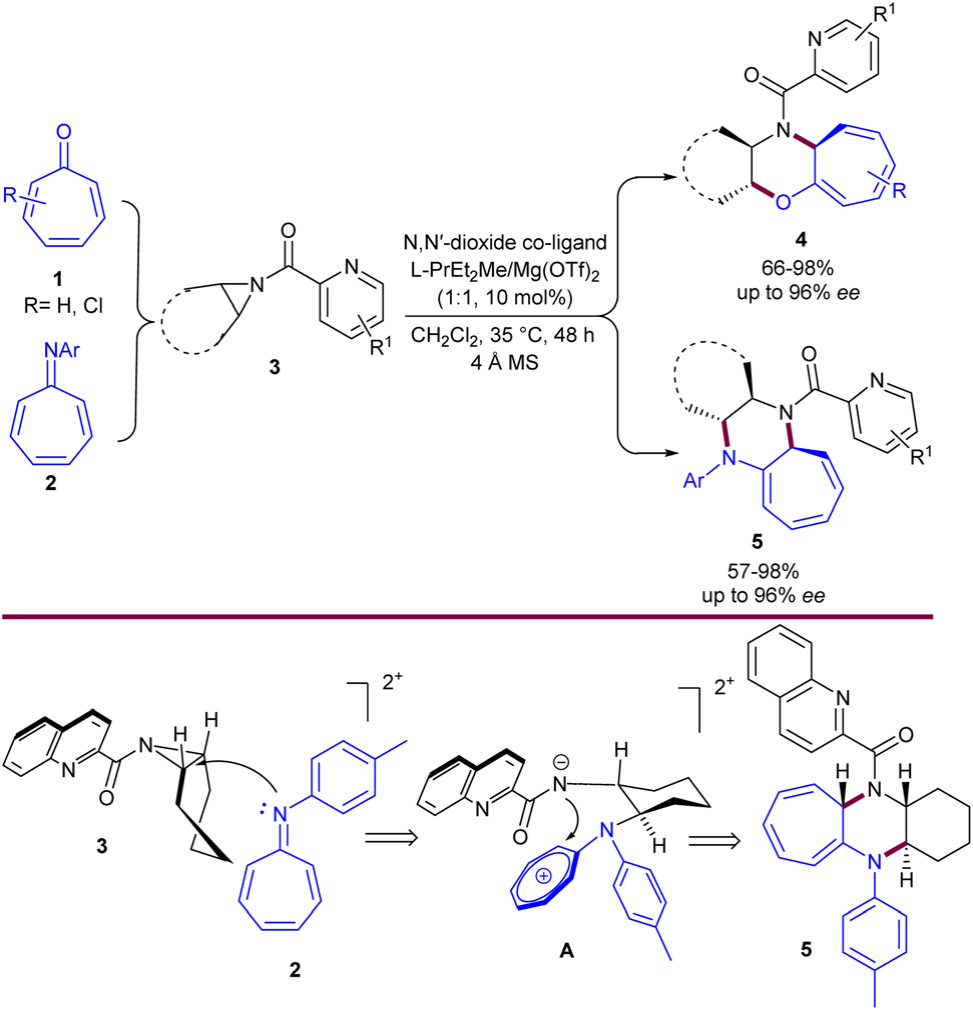
Mg-catalyzed (8 + 3)-cycloaddition of tropone or azaheptafulvenes with *meso*-aziridines.

A highly enantioselective magnesium-catalyzed system for the (8 + 3)-cycloaddition of tropone 1 or azaheptafulvenes 2 with *meso*-aziridines 3 was developed by Liu, Feng, and coworkers (Scheme 1).^33^ This desymmetrization/annulation process proceeded in the presence of chiral *N*,*N*′-dioxide/Mg(OTf)_2_ complex (1 : 1, 10 mol%), in DCM at 35 °C. A new series of tricyclic heterocycles containing a cycloheptatriene motif 4, 5 was synthesized in up to 98% yield, 96% ee, and >19 : 1 dr. The aziridine 3 bound the Mg with both the pyridine nitrogen and the carbonyl oxygen group. So, the cyclohexenyl ring of aziridine is oriented downwards to have the lowest spatial repulsion with the top-right amide of the ligand. At this time, the nitrogen of the azaheptafulvene 2 attacked from behind the aziridine ring at the outer carbon center, producing *trans*-cyclohexane-1,2-diamino intermediate after ring-opening. Subsequent annulation yielded the final product 5. Moreover, the synthetic application of this method was demonstrated with the gram-scale synthesis of the final product (1.12 g, 90% yield, 91% ee).

#### 2.1.2. Ag-catalyzed cycloaddition reactions involving tropone

**Scheme 2:**
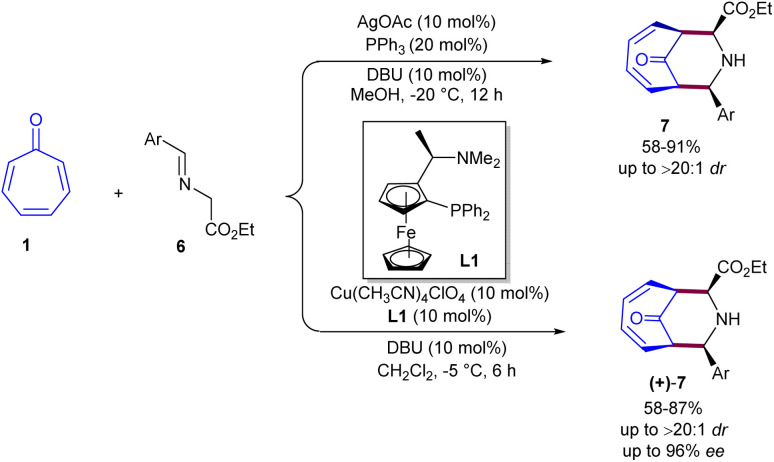
(6 + 3)-Cycloaddition between tropone and azomethine ylides.

**Scheme 3:**
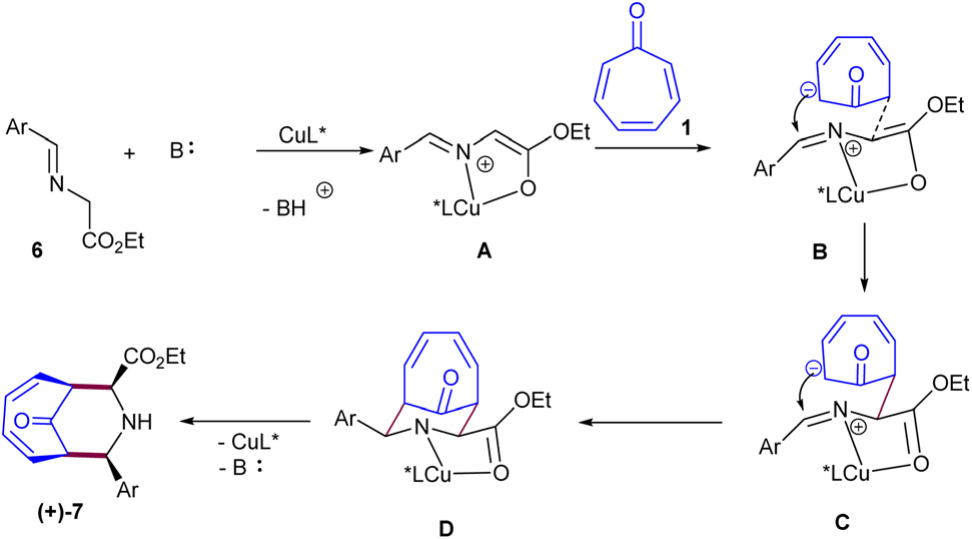
Proposed mechanism or asymmetric (6 + 3)-cycloaddition of tropone and azomethine ylides.

**Scheme 4:**
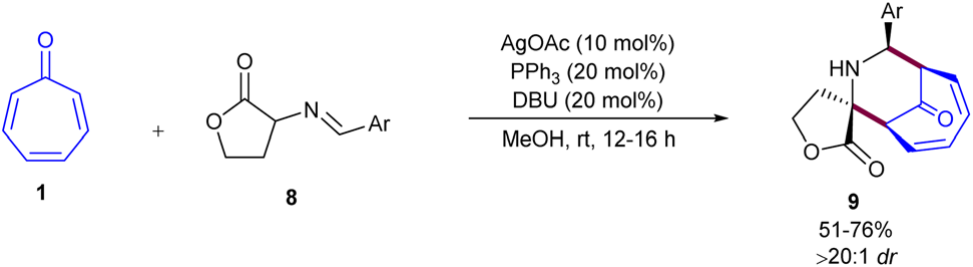
(6 + 3)-Cycloaddition of tropone and azomethine ylides.

In 2014, Honglei Liu *et al.* reported the first metal-catalyzed (6 + 3)-cycloaddition of tropone 1 with azomethine ylides 6 (Scheme 2).^34^ Two metal salts, Ag(i) and Cu(ii), were tested in the catalytic cycloaddition of tropone with azomethine ylides towards piperidine-fused bicyclic heterocycles. A silver acetate catalyst, in combination with a triphenyl phosphine ligand, can catalyze the diastereoselective (6 + 3)-cycloaddition of tropone with azomethine ylides. The use of a chiral ferrocenylphosphine in the copper-catalysis system enabled the achievement of final products with good to excellent enantioselectivities. The catalytic asymmetric (6 + 3)-cycloaddition reaction was suggested to occur through the formation of carboxyl enolate cuprous salt A, resulting from the abstraction of acidic hydrogen by the base under copper catalysis. This active species rapidly underwent nucleophilic addition to tropone towards zwitterion C through the transition state B. The steric crowding at the front side of A guided the approach of tropone from the backside. Subsequent intramolecular nucleophilic addition to the *si* face of the imine gave intermediate D, which was protonated to yield product 7 with release of the Cu(i) species and the base for the next catalytic cycle (Scheme 3). Huang's research group reported (6 + 3)-cycloaddition reaction between tropone 1 and azomethine ylides 7 (Scheme 4).^35^ Various azomethine ylides prepared from homoserine lactone and aryl aldehydes with both electron-donating and electron-withdrawing substituents smoothly reacted with tropone at room temperature to yield tricyclic spiropiperidines in moderate to excellent yields (51–92%) with excellent diastereoselectivities (>20 : 1 dr). The position of the substituent on the aryl ring in the azomethine ylides had no notable influence on the yield and diastereoselectivity. Azomethine ylides prepared from 2-CH_3_, 4-OCH_3_, and 2-F substituted benzaldehydes showed moderate reactivity. The 1-naphthyl-substituted azomethine ylide exhibited good reactivity, yielding the corresponding product in 67% yield with excellent diastereoselectivity.

#### 2.1.3. Ni-catalyzed cycloaddition reactions involving tropone

**Scheme 5:**
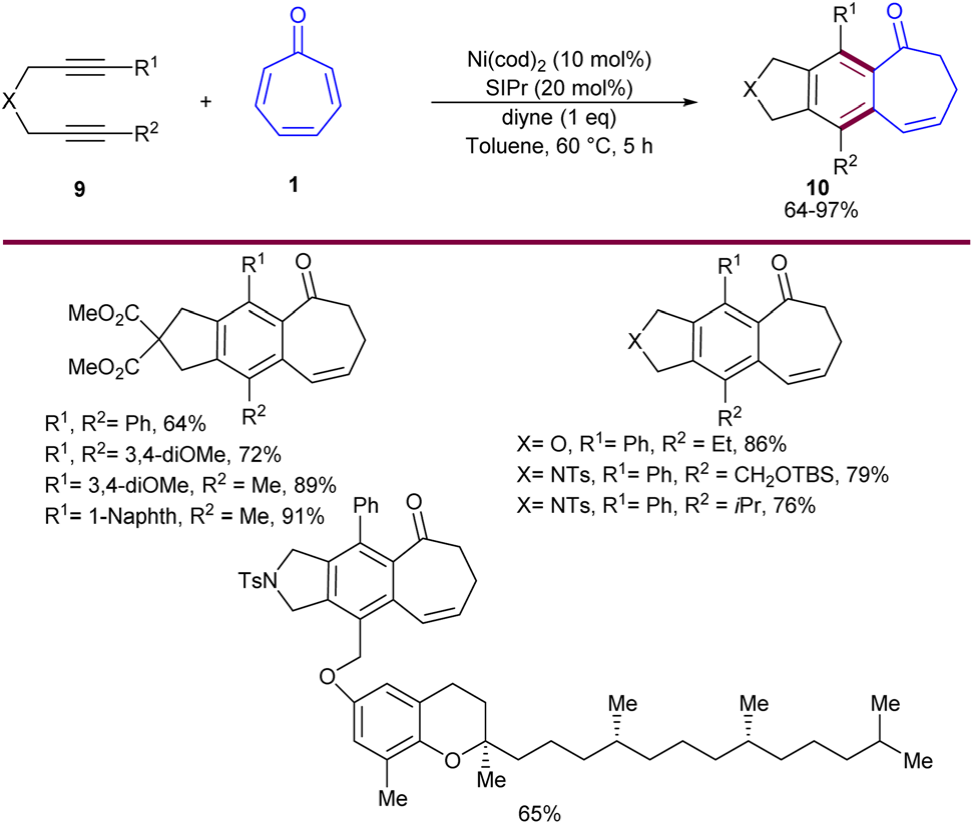
Ni(NHC)-catalyzed cycloaddition of diynes and tropone.

**Scheme 6:**
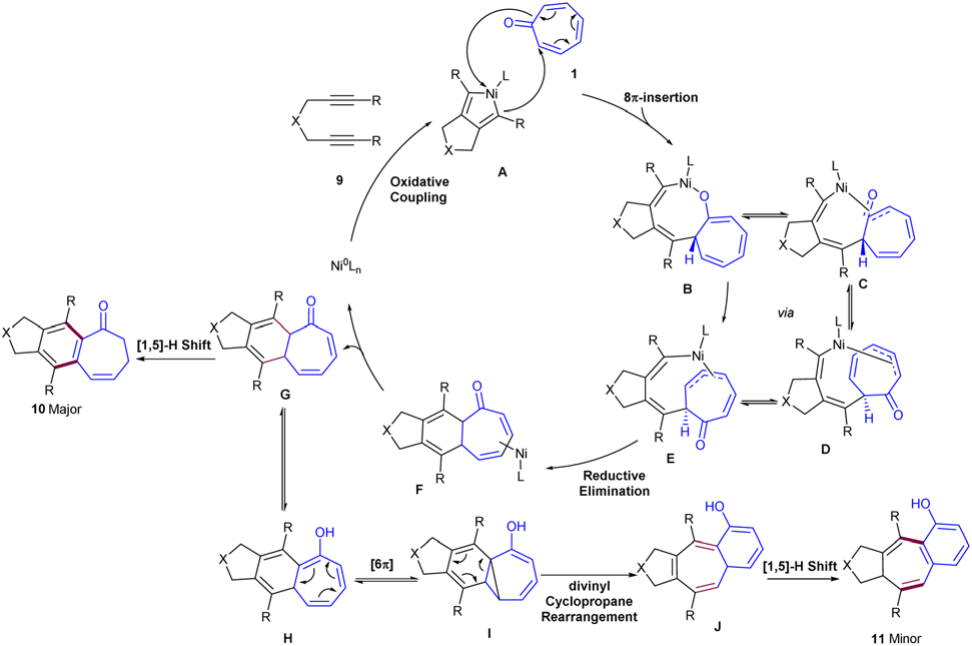
Catalytic cycle for Ni(NHC)-catalyzed cycloaddition of diynes and tropone.

**Scheme 7:**
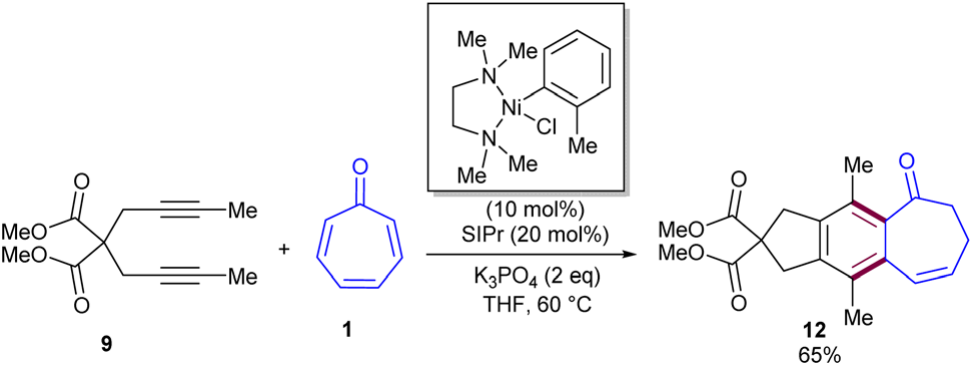
[(TMEDA)Ni(*o*-tolyl)Cl]-catalyzed cycloaddition of tropone with diynes.

Nickel-catalyzed cycloaddition of tropone 1 with diynes 9 was reported by Louie and co-workers in 2014 (Scheme 5).^36^ Various diynes were efficiently coupled with tropone, affording [5-6-7] fused tricyclic products. The reaction had broad substrate scope with respect to diynes, and excellent regioselectivities were observed in the case of unsymmetrical diynes. In the mechanism, nickel can selectively incorporate into the C–C π-bond of tropone, forming both major and minor products. The reaction began by the homo-oxidative coupling of diyne on Ni(0) to obtain Ni(ii)-cyclopentadiene A that underwent 8π insertion of tropone 1 to generate a seven-membered ring complex IB, followed by isomerization to form intermediate C. Then, C isomerized to intermediate D, which in turn isomerized to intermediate E, which, upon reductive elimination, gave intermediate F. Next, F was converted into intermediate G, followed by [1,5]-H shift to form major product 10. On the other hand, G can move through another pathway involving tautomerization to form cycloheptatrienol H, followed by 6π-electrocyclization to afford bis(divinyl)-cyclopropane I. This intermediate can either revert to H or irreversibly rearrange to [5-7-6]-fused intermediate J through divinyl cyclopropane rearrangement. Further sigmatropic shifts in J afforded minor product 11 (Scheme 6). The authors mentioned that the presence of a small amount of water could catalyze this sigmatropic shift step *via* the bridging of one or multiple water molecules. A new air-stable, crystalline solid [(TMEDA)Ni(*ortho*-tolyl)Cl] was utilized for the cycloaddition of tropone 1 with diynes 9 (Scheme 7).^37^ Preliminary mechanistic studies showed that [(TMEDA)Ni(*ortho*-tolyl)Cl] can be activated by either Ni–C or Ni–Ni transmetalation.

**Scheme 8:**
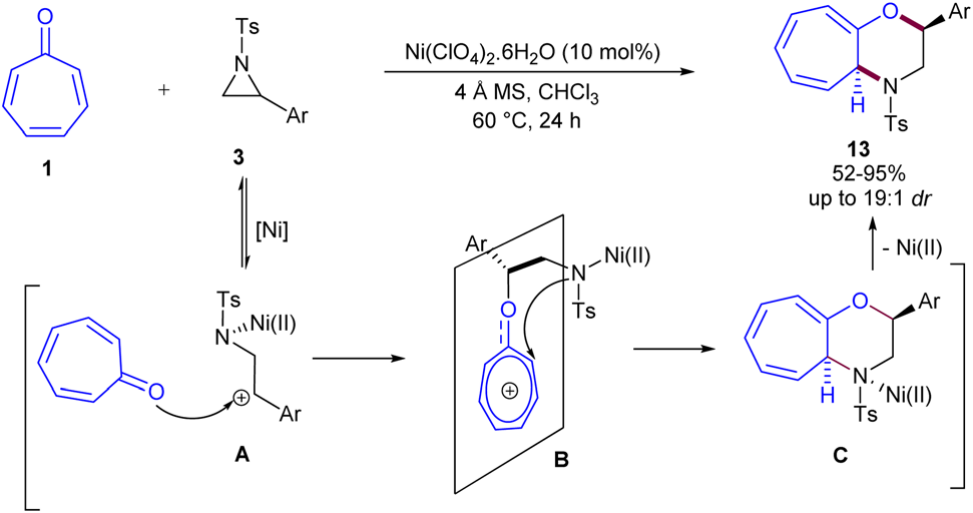
Ni-catalyzed (8 + 3)-cycloaddition of tropone with 2-aryl-*N*-tosylaziridines.

The (8 + 3)-cycloaddition of tropone and 2-aryl-*N*-tosylaziridines can be catalyzed in the presence of a nickel(ii) catalyst (Scheme 8).^38^ A wide range of aziridines bearing both electron-rich and electron-deficient aryl moieties reacted well with tropone, affording various 4-tosyl-2,3,4,4*a*-tetrahydrocyclohepta[*b*]–[1,4]oxazines in moderate to excellent yields with good diastereoselectivities. To investigate the mechanism, the authors conducted a reaction of an enantiopure aziridine with tropone, resulting in a racemic product, which confirmed the opening of the aziridine ring during the reaction. Therefore, the mechanism involves a Ni(ii)-catalyzed ring opening of aziridine 3, generating intermediate A, followed by a nucleophilic attack of tropone 1 on the carbocation from the top of the tropone plane, as the Ar group blocks the bottom of the tropone. This produced intermediate B with good diastereoselectivity. Another nucleophilic attack led to intramolecular cyclization towards intermediate C, which, upon removal of the Ni catalyst, resulted in the liberation of the final product 13. Furthermore, the large-scale synthesis of the product (1.55 g, 82%, 13 : 1 dr) and the reduction of the tropone ring demonstrated the synthetic utility of this method.

#### 2.1.4. Rh-catalyzed cycloaddition reactions involving tropone

**Scheme 9:**
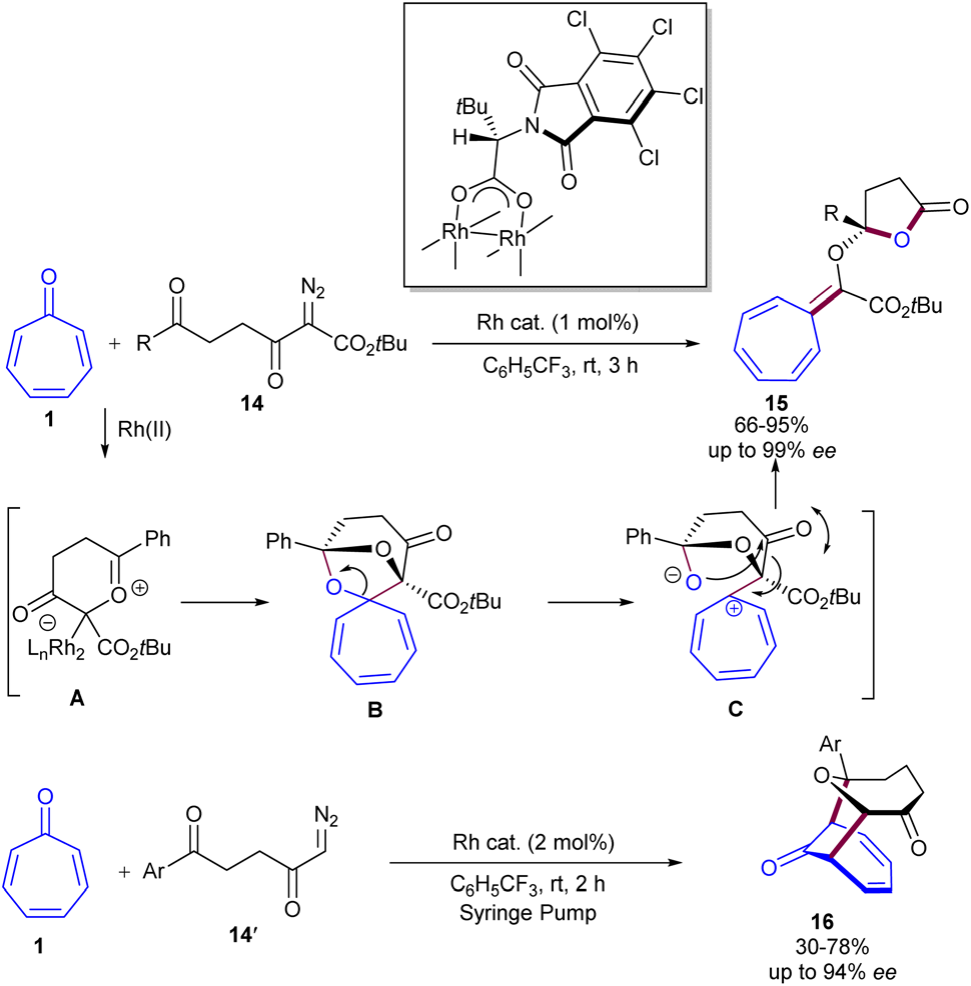
Rh-catalyzed enantioselective 1,3-dipolar cycloaddition of tropone and carbonyl ylides.

In 2015, Sandip Murarka *et al.* reported Rh-catalyzed enantioselective (3 + 2)-cycloaddition reaction between tropone 1 and α-diazoketone-derived carbonyl ylides 14 (Scheme 9).^39^ A chiral rhodium complex was utilized in the reaction of tropone 1 and diazodiketoester 14 to construct lactone 15 with complete diastereoselectivity (>20 : 1 dr) and excellent enantioselectivity (up to 99% ee). Using the same rhodium catalyst, bridged polyheterocyclic compounds 16 were constructed through (6 + 3)-cycloaddition of the conjugated triene system of tropone 1 as electrophile with the carbonylylide derived from α-diazoketone 14′ as dipoles. The reactivity difference arises from the different HOMO/LUMO energy levels of carbonyl ylides derived from diazodiketoesters and α-diazoketones. In general, the reaction proceeded through (3 + 2)-cycloaddition of a carbonyl ylide 14 with the keto group of tropone 1 to form spirocyclic intermediate A. Then, A was converted into zwitterion B, followed by intramolecular (3 + 2)-cycloaddition and rearrangement to generate lactone 15.

#### 2.1.5. Au-catalyzed cycloaddition reactions involving tropone

**Scheme 10:**
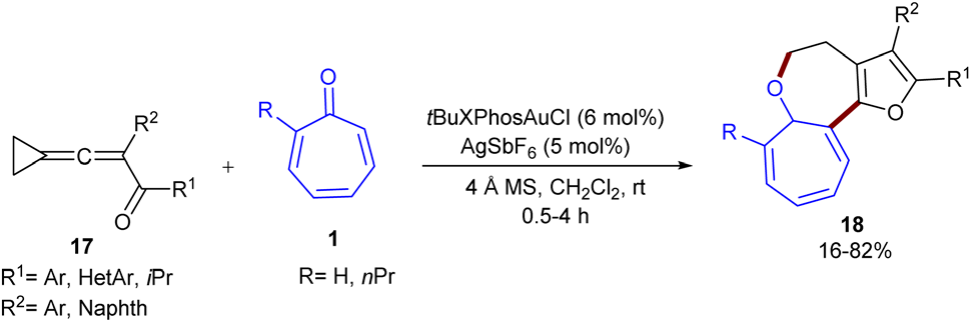
Au-catalyzed reaction of tropone and cyclopropyl-tethered allenyl ketone.

**Scheme 11:**
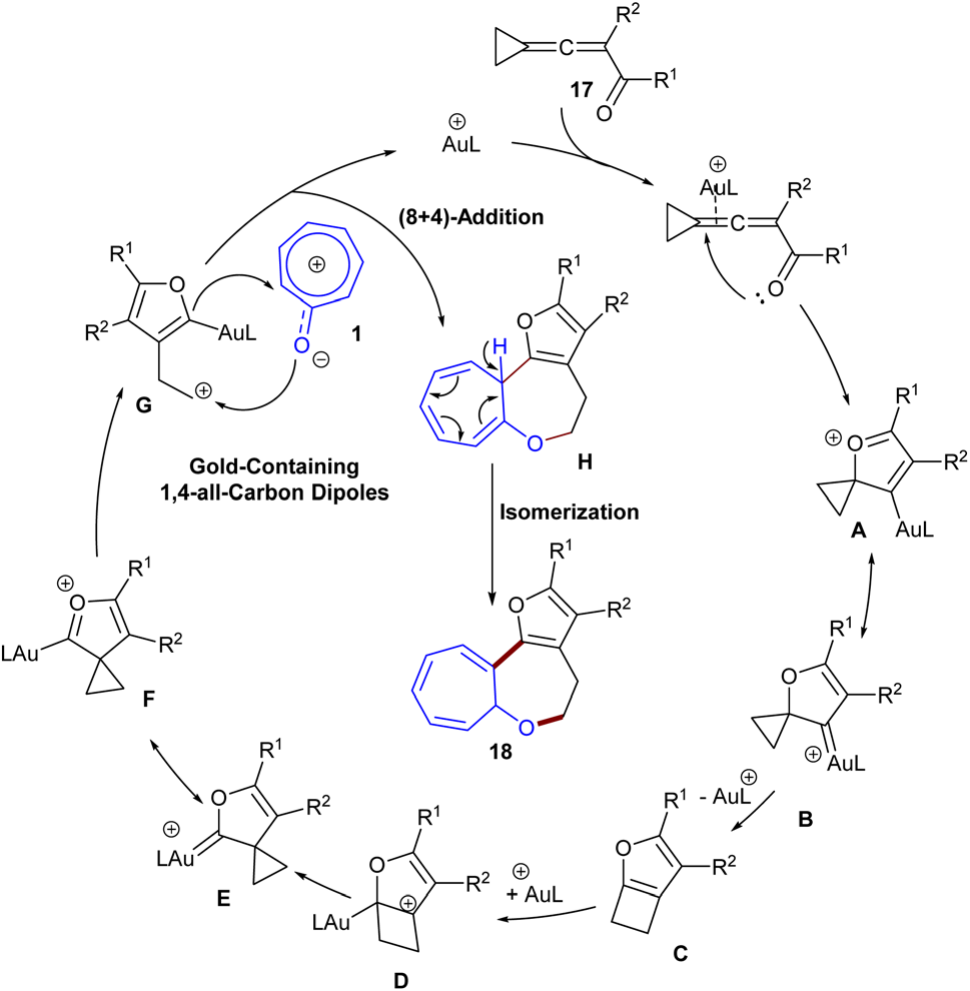
Catalytic cycle for Au-catalyzed reaction of tropone and cyclopropyl-tethered allenyl ketone.

In 2020, Miao's research team developed a gold catalysis system for the (8 + 4)-annulation reaction between tropone 1 and cyclopropyl-tethered allenyl ketone derivatives 17 (Scheme 10).^40^ A gold-containing 1,4-allcarbon dipole derived from allenyl ketone bearing a cyclopropyl moiety was found to be a key intermediate in the high-order (8 + 4)-cycloaddition with tropone, delivering 7,7,5-fused tricyclic frameworks in low to high yields (16–82%) with complete selectivity. According to the mechanism, the reaction begins with the π-activation of allene by the cationic Au(i) catalyst, followed by nucleophilic cyclization to the spirocyclic oxonium ion A and its resonance structure, the gold-carbene form B. Then, B underwent ring-expansion and proto-demetalation to generate the furan-fused cyclobutene C. The re-addition of cationic gold species to a highly strained C=C bond of C led to the cationic furan D. Subsequent ring contraction in D resulted in the gold carbene E and spirocyclic oxonium ion F as resonance species. Ring opening of F generated the gold-containing 1,4-all-carbon dipole G, which moved through intermolecular (8 + 4)-cycloaddition with tropone 1*via* either a stepwise or a concerted route to furnish intermediate H. The obtained intermediate underwent C=C bond isomerization to deliver product 18 (Scheme 11). The gram-scale synthesis of the product resulted in 1.12 g, 65% yield. The direct reduction of the tropone ring yielded the hydrogenation product in 70% yield, which, after a (4 + 2)-cyclization with *in situ* generated benzyne, provided an *O*-bridged polycyclic compound.

**Scheme 12:**
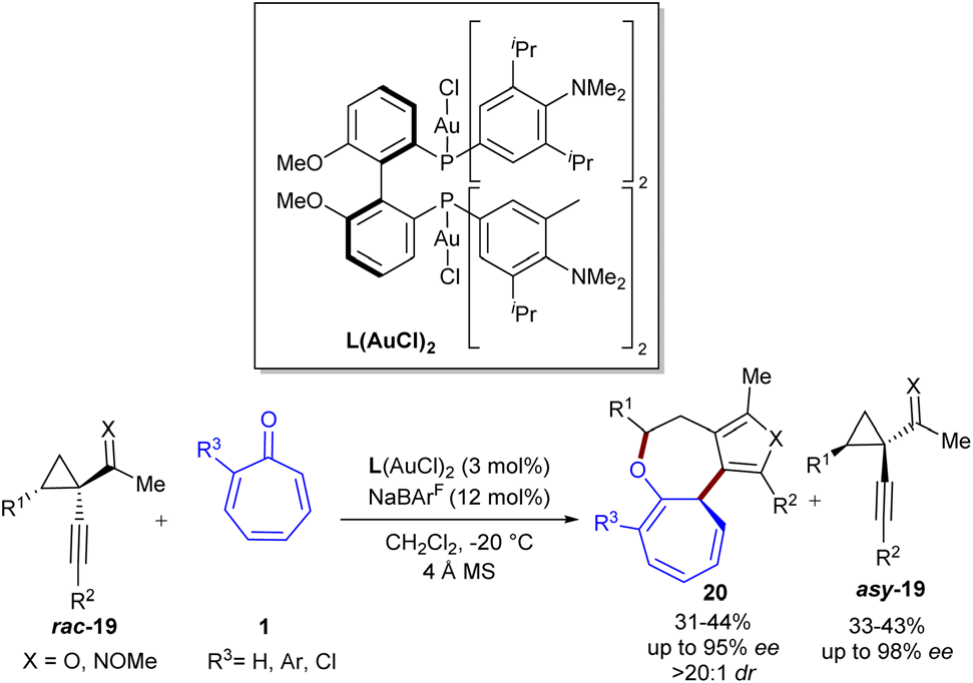
Au-catalyzed (8 + 4)-cycloaddition of tropone with 1-(1-alkynyl)cyclopropyl ketones.

Au-catalyzed regio-, stereo-, and enantioselective (8 + 4)-cycloaddition of tropone 1 with 1-(1-alkynyl)cyclopropyl ketones 19 was reported by Wang *et al.* in 2024 (Scheme 12).^41^ A variety of tropone and 2-halo/aryl tropones smoothly participated in the reaction with cyclopropyl ketones bearing aryl/heteroaryl/cyclohexenyl, producing highly functionalized cyclohepta[*b*]furo[3,4-*d*]oxepines 20 in satisfactory yields with >20 : 1 dr, and up to 95% ee. The reaction of oxime-containing substrate 2 with tropone was also successful under this gold catalysis system, yielding chiral [5.5.0] bicyclic product containing a pyrrole moiety in 31% yield and 73% ee. Additionally, a kinetic resolution study using racemic 1-(1-alkynyl)cyclopropyl ketones *asy*-19 was performed (*s* factor up to 104). The synthetic utility of this method was demonstrated by the gram-scale synthesis of the product (0.76 g, 40% yield, 92% ee), the hydrogenation of the troponyl ring, and the (4 + 2)-cyclization of the furan unit with *in situ* generated benzyne. Additionally, the [1,5]-H shift of some products was performed in the presence of PTSA at room temperature, yielding the corresponding products in 61–71% yields and 80–88% ee.

#### 2.1.6. Pd-catalyzed cycloaddition reactions involving tropone

**Scheme 13:**
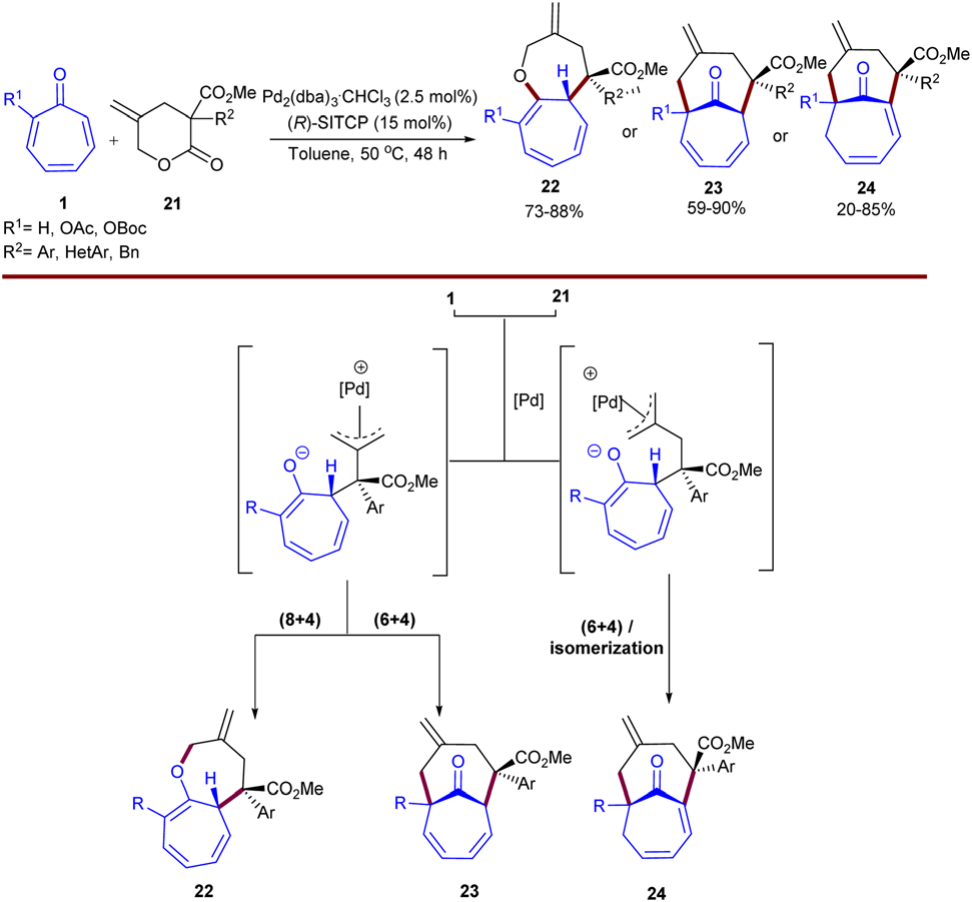
Pd-catalyzed reaction of tropones with γ-methylidene-δ-valerolactones.

In 2020, Lan's research team reported the first example of Pd-catalyzed cycloaddition reaction of tropones 1 with γ-methylidene-δ-valerolactones 21 (Scheme 13).^42^ Merging of a palladium(0) complex with a chiral phosphine ligand can constitute an efficient diastereo- and enantioselective catalytic system for the direct synthesis of [5.5.0] and [4.4.1] bicyclic compounds. Mechanistic studies and DFT calculations revealed that two possible key diastereomeric intermediates could be generated, leading to three classes of medium-sized bicyclic compounds. One intermediate can undergo either O- or C-allylation, providing [5.5.0] or [4.4.1] bicyclic compounds 22, and 23 through (8 + 4)- or (6 + 4)-cycloaddition reaction, respectively. In contrast, another conformation of diastereomer afforded bridgehead alkene-containing bicyclo[4.4.1] compounds 24*via* (6 + 4)-cycloaddition/unconventional alkene isomerization. By controlling the reaction conditions, the authors were able to increase the yield of each product as the major product.

**Scheme 14:**
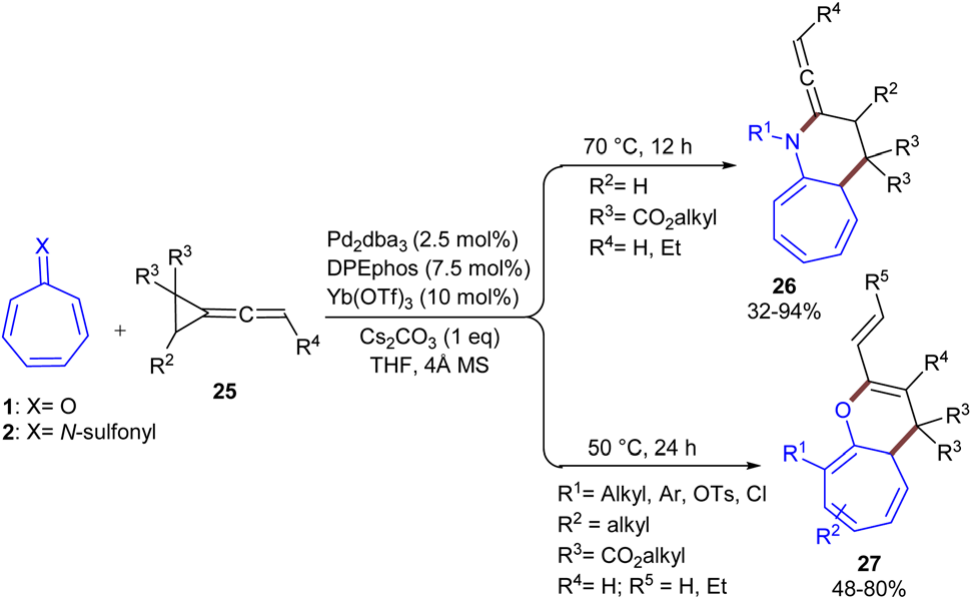
Pd/Yb-catalyzed reaction of tropones with vinylidenecyclopropane-diester.

**Scheme 15:**
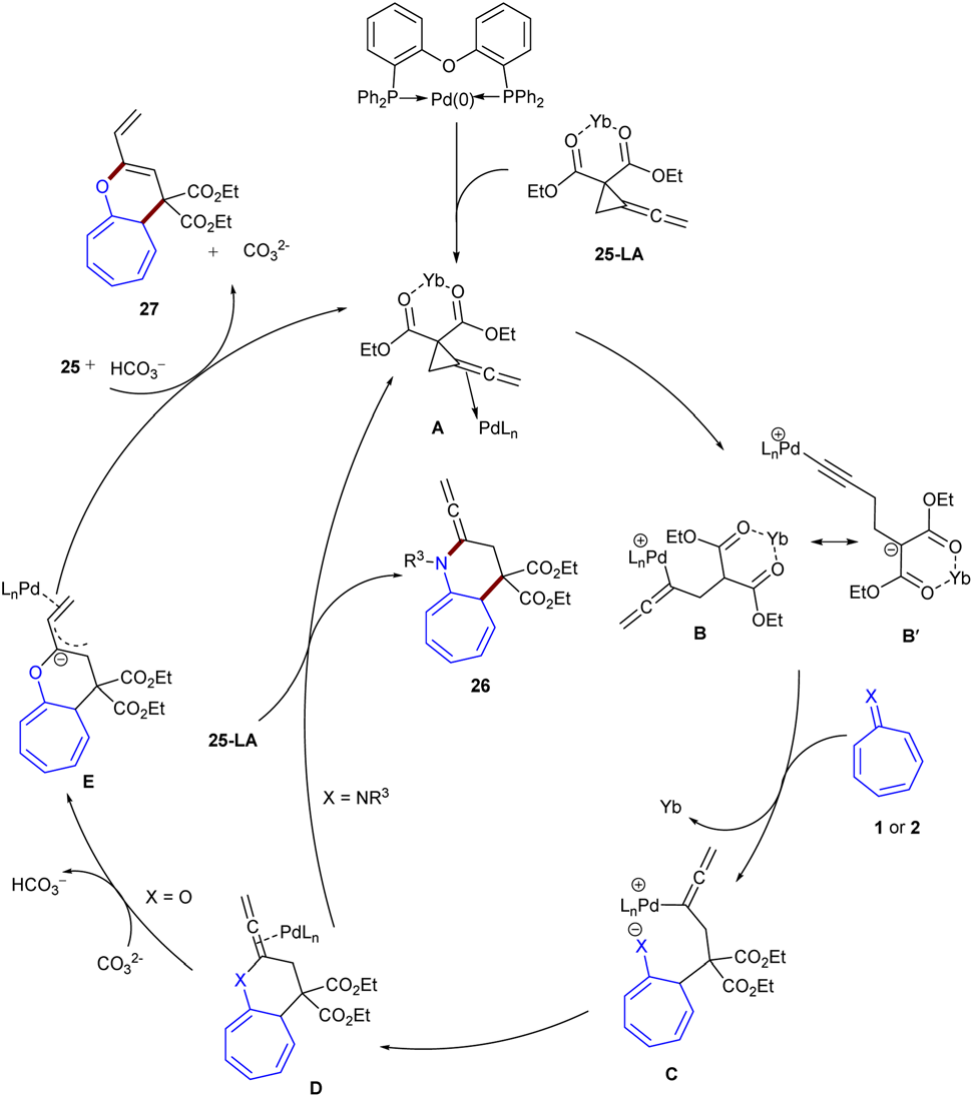
Catalytic cycle for Pd/Yb-catalyzed reaction of tropones with vinylidenecyclopropane-diester.

**Scheme 16:**
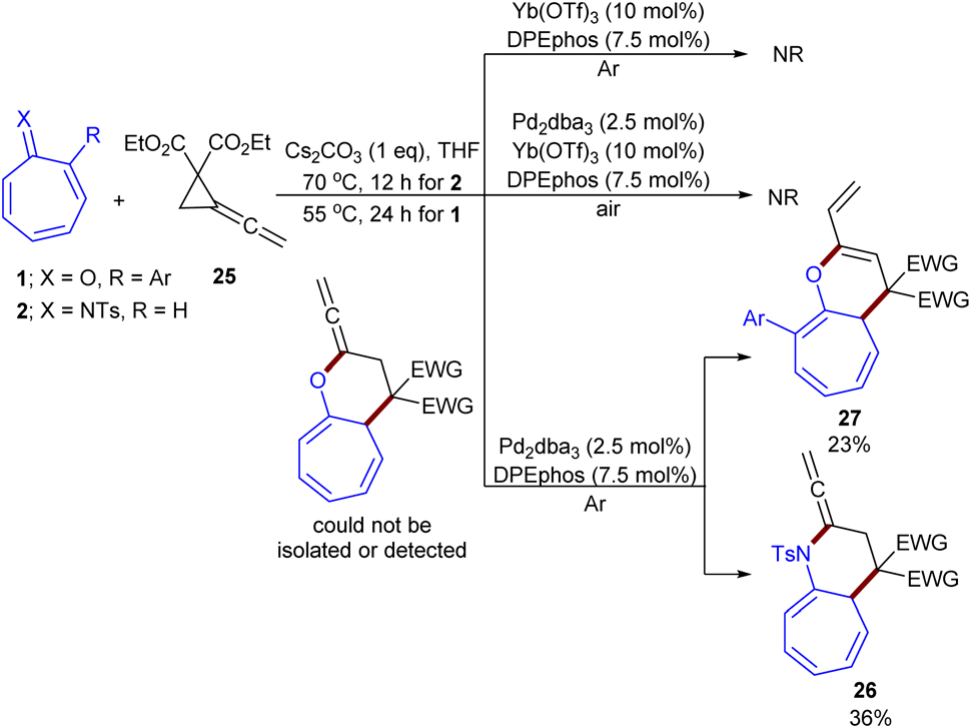
Pd-catalyzed reaction of tropones with vinylidenecyclopropane-diester.

In 2024, Shi, Wei and co-workers established the (8 + 3)-cycloaddition of tropones 1 or tropsulfimides 2 with vinylidenecyclopropane-diester 25 (Scheme 14).^43^ In this reaction system, the nature of tropone derivatives can influence on the product formation; decahydro-1*H*-cyclohepta[*b*]pyridines bearing an allene unit 26 were obtained when X = N, whereas decahydro-1*H*-cyclohepta-[*b*]pyrans 27 were the main product when C=O. The structure of zwitterionic propargyl palladium intermediates was confirmed with the help of DFT calculations and HRMS experiment. Thereby, a rational mechanism was suggested for this cycloaddition. First, Pd(0) coordinated to vinylidenecyclopropane-diester 25 to generate complex A. This complex underwent oxidative addition at the electron-deficient C–C site of the cyclopropyl motif, yielding intermediate B. The isomerization of B produced zwitterionic propargyl palladium species B′, which underwent (8 + 3)-cycloaddition with tropsulfimide 25 or tropone 1 to obtain intermediate C. Subsequent intramolecular nucleophilic addition yielded intermediate D. When X = NSO_2_R, ligand exchange by 25 afforded product 26 and regenerated A. For X = O, proton transfer driven by the carbonate anion occurred to form allyl anionic intermediate E, which underwent protonation of E on another side of the allyl anion with the bicarbonate anion, delivering product 27, and the ligand exchange with 25 regenerated the Pd complex A to fulfill the catalytic cycle (Scheme 15). It was found that palladium catalyst, Lewis acid, and the base are all necessary for the reaction to proceed. In another study, this research group investigated the reaction of tropones/tropsulfimides with vinylidenecyclopropane-diester in the presence and absence of the Lewis acid Yb(OTf)_3_ to determine the impact of the Lewis acid on the promotion of oxidative addition (Scheme 16).^44^ By studying DFT results and HRMS experiments, they could confirm the position of C–C oxidative addition cleavage and the positive impact of the Lewis acid Yb(OTf)_3_ in promoting oxidative addition.

#### 2.1.7. Mo-catalyzed cycloaddition reactions involving tropone

**Scheme 17:**
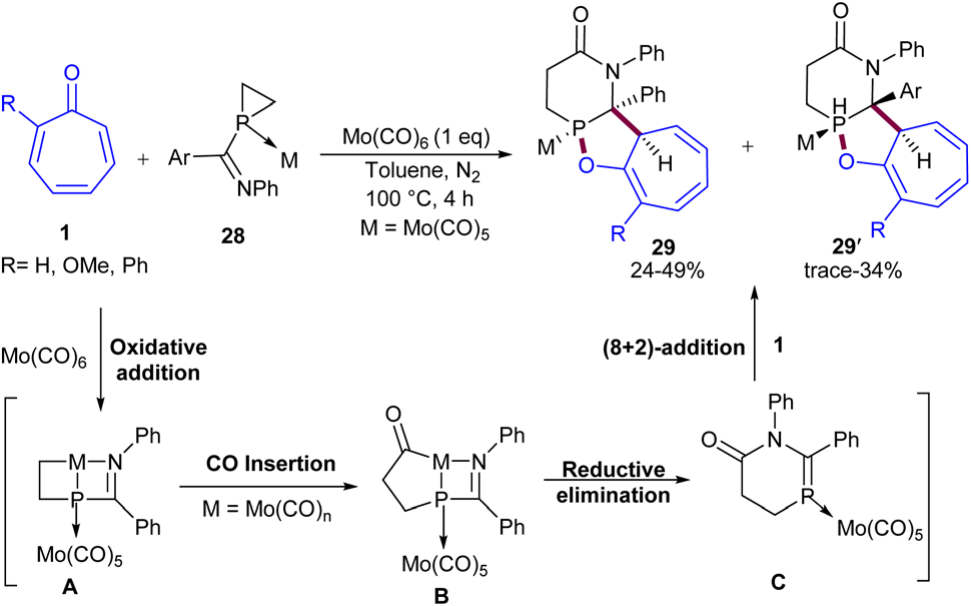
Mo-catalyzed (5 + 1)/(8 + 2)-cycloadditions of phosphiranes and tropones.

In 2022, Tian and co-workers disclosed a tandem (5 + 1)/(8 + 2)-cycloaddition reaction between tropones 1 and phosphiranes 28 (Scheme 17).^45^ Tropone, 2-phenyl tropone, and 2-methoxy tropone were well incorporated in the reaction with 1-iminylphosphiranes bearing *N*-aryl moieties. The highly reactive C=P bond, derived from the carbonylative ring expansion of 1-iminylphosphirane complexes, enabled high-order (8 + 2)-cycloaddition of tropones, leading to structurally diverse 6,5,7-fused tricyclic scaffolds in satisfactory yields with excellent site selectivity. As shown in the mechanism, the oxidative addition of Mo(0) to the C–P bond of the strained phosphirane complex B resulted in the cyclocarbonylation of 1-iminylphosphirane. Sequential CO insertion and reductive elimination resulted in azaphosphacyclohexone complex C. The following (8 + 2)-cycloaddition between the C=P bond of 5 and tropone delivered 29 and 29′ as *endo*/*exo* isomers.

### 2.2. Metal-free cycloaddition reactions involving tropone

#### 2.2.1. Organocatalyst-catalyzed cycloaddition reactions involving tropone

**Scheme 18:**
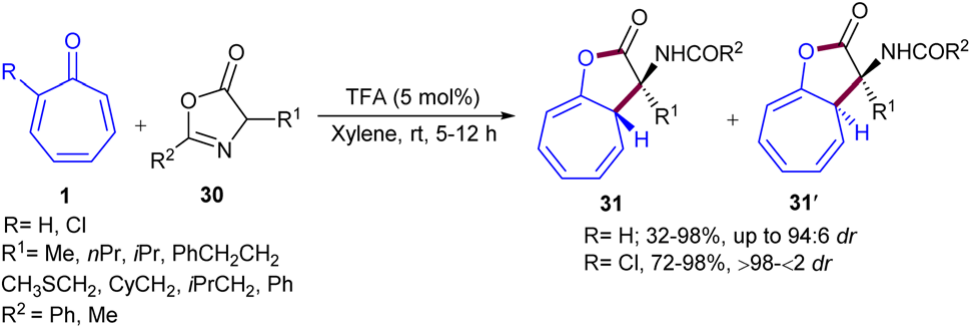
(8 + 2)-Formal cycloaddition reactions of tropones with azlactones.

**Scheme 19:**
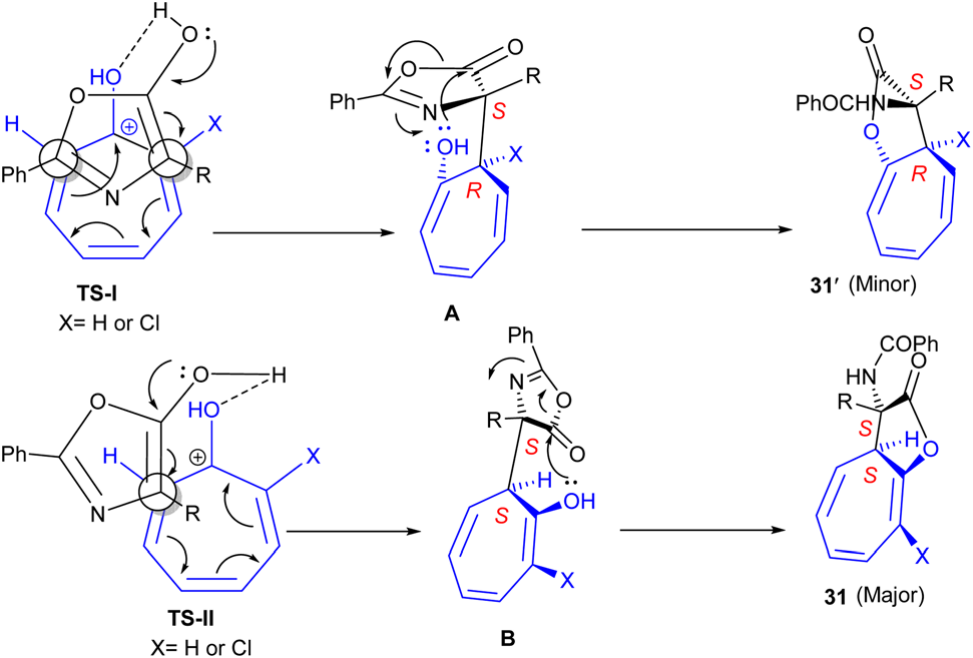
Possible transition states in the reaction of protonated tropones with azlactones.

A Brønsted acid catalyst was used for tropones 1 with azlactones 30 to construct dihydro-2*H*-cyclohepta[*b*]furan skeleton 31 (Scheme 18).^46^ In this reaction, both tropone, as an electrophile, and azlactone, as a nucleophile, were activated by the action of a Brønsted acid catalyst. A hydrogen bond was formed between the OH group of the enol and the oxygen atom of the protonated tropone, which could be the origin of the stereoselectivity in the reaction. The authors assumed two transition states (TS-I and TS-II), differing in the diastereotopic carbon atom (C-α in TS-I or C-α in TS-II), which were attacked by the enol nucleophile. TS-I was less stable compared to TS-II due to steric interactions, which could explain the predominance of the S*,S* isomer in the reaction mixture. The evolution of the obtained intermediates by intramolecular ring-opening of azlactone liberated products 31 (major) and 31′ (minor) (Scheme 19). It was found that the presence of a substituent at the electrophilic α-carbon of tropone has a significant impact on chemoselectivity because it produced an additional destabilization of A (with the Cl/R interaction instead of H/R) without affecting B, which favored 31 concerning 31′. In addition, the presence of the Cl group at the α-carbon of tropone could generate quaternary amino acids with the tropone ring at the α-carbon. Since α-chlorotropone was less reactive than tropone and full conversion occurred after 24 hours, the chemoselectivity could be controlled entirely, affording the diastereomerically pure products from the attack at the unsubstituted α-position. Additionally, further ring-opening of the obtained product in the presence of various nucleophiles can result in the formation of α,α-disubstituted α-amino acid derivatives.

**Scheme 20:**
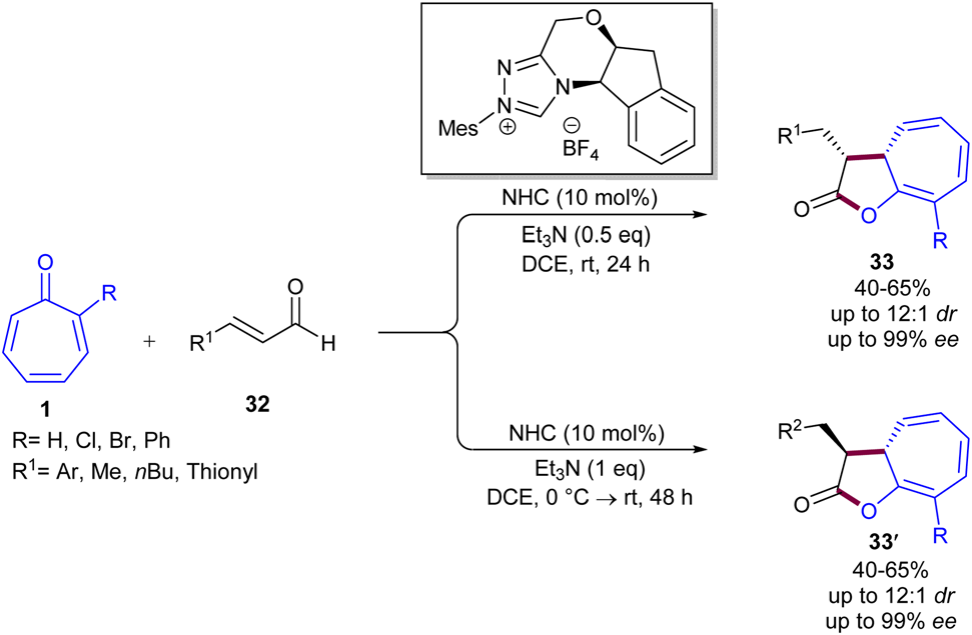
NHC-catalyzed (8 + 2)-cycloaddition of enals with tropones.

**Scheme 21:**
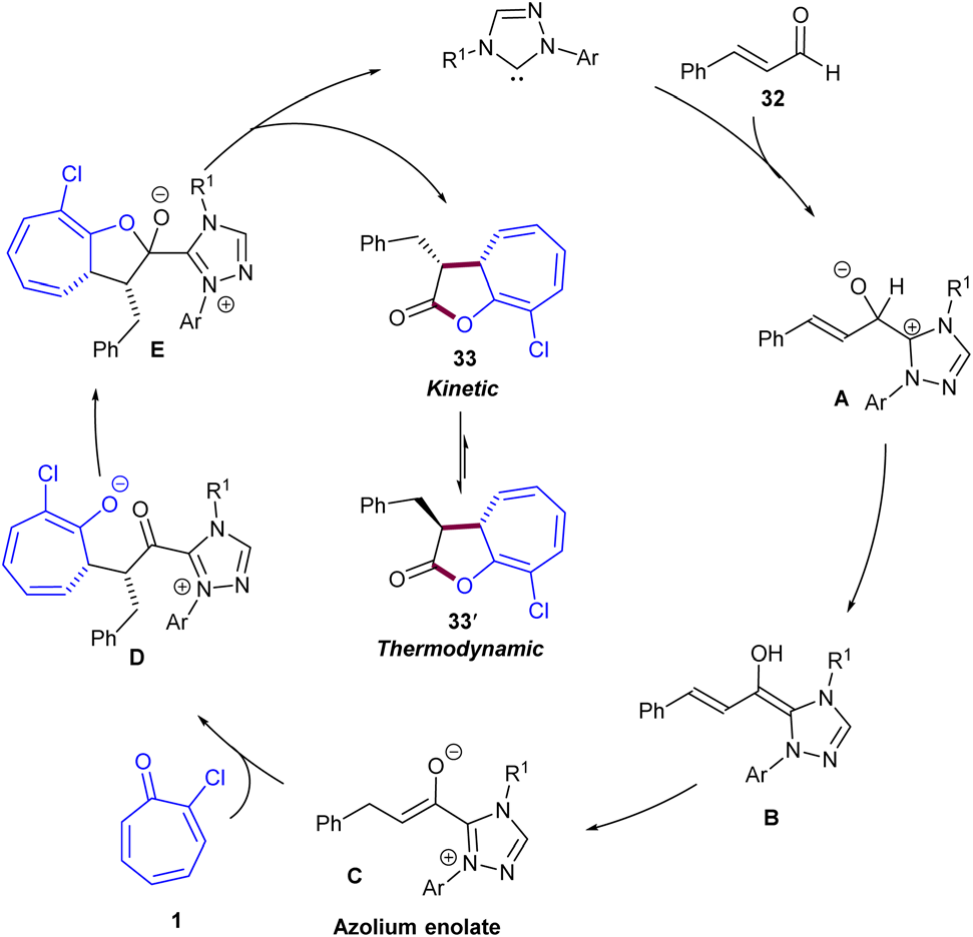
Credible catalytic cycle for NHC-catalyzed (8 + 2)-cycloaddition of enals with tropones.

**Scheme 22:**
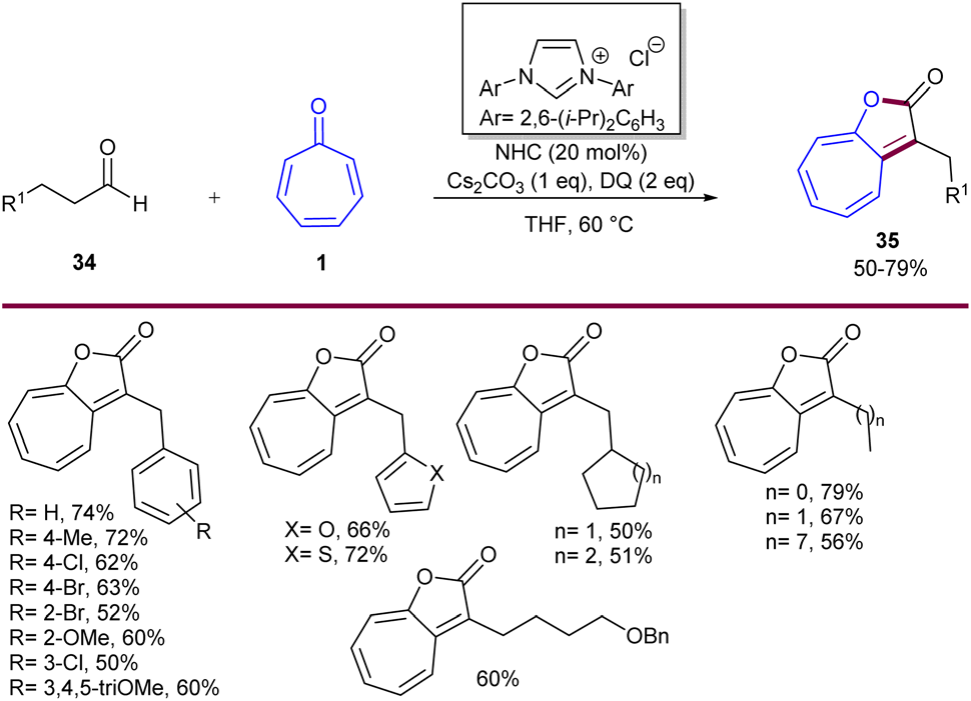
NHC-catalyzed (8 + 2)-cycloaddition of aldehydes with tropones.

Maseras and his colleagues employed *N*-heterocyclic carbene catalysts for asymmetric (8 + 2)-cycloadditions of tropones 1 with enals 32 (Scheme 20).^47^ This is a classic example of using NHC catalysts in (8 + 2)-cycloadditions of tropones, which provides access to a kinetic *cis*-cycloadduct that can be epimerized to its trans analogue by using an excess amount of base and a longer reaction time. Besides, the obtained cycloadducts can be derivatized by hydrogenation or methanolysis. According to DFT calculation results, a credible mechanism was proposed for this transformation, which involved the interaction of NHC with enal 32 to form the Breslow intermediate A, followed by the conversion of A to azolium anolate C*via*B. After that, C underwent a rapid and irreversible 1,8-addition to tropone 1 through transition state T4 to form D, which determined both the enantio- and the diastereoselectivity of the process. Then, D converted into E, followed by the release of product 4 and the regeneration of the NHC catalyst. The obtained cycloadduct can also undergo NEt_3_-catalyzed epimerization, leading to the thermodynamically stable trans diastereomer. Since only the *Z*-enolate in intermediate C was energetically available, the cycloaddition only led to the products with (33-*R*,33-*S*) and (33-*R*,33-*R*) configurations, depending on the face (*Re* or *Si*, respectively) of the tropone involved in the reaction (Scheme 21). Another example of the use of NHCs in the (8 + 2)-cycloaddition of tropones 1 was reported by Ye and co-workers (Scheme 22).^48^ For this purpose, aldehydes 34 served as a reactant in the annulation with tropones to construct cycloheptanefused furanones 35. A similar mechanism to Maseras' reaction was suggested, involving the formation of the Breslow intermediate, and the enolate intermediate, followed by intramolecular acylation and liberation of the product after aromatization and regeneration of the NHC catalyst. The production of the gram-scale synthesis (1.29 g, 80%) and hydrogenation of the product demonstrated the synthetic application of this method.

**Scheme 23:**
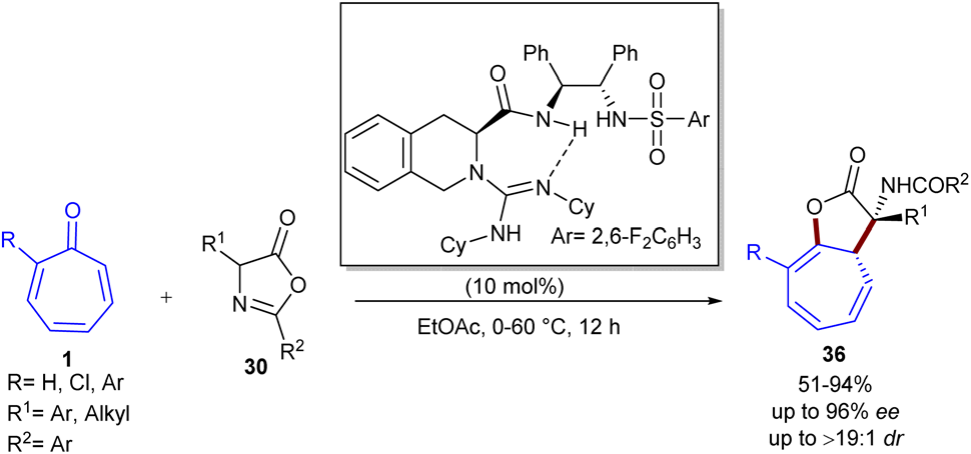
Organocatalytic (8 + 2)-cycloaddition of tropones with azlactones.

**Scheme 24:**
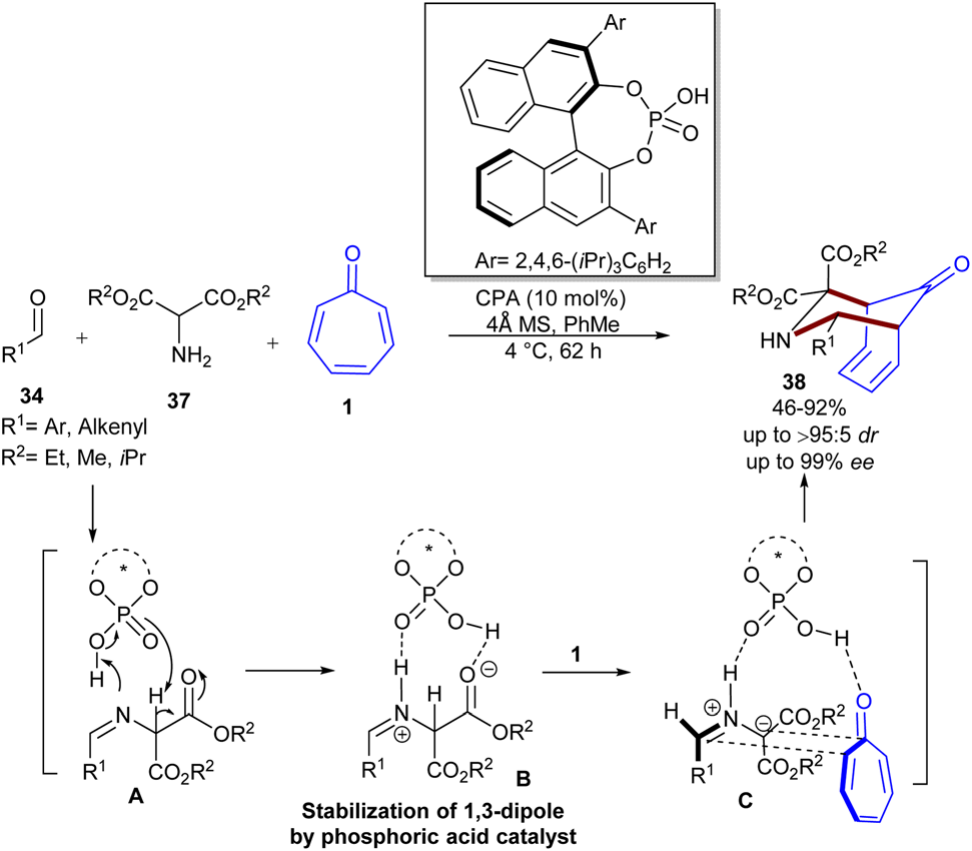
(6 + 4)-Cycloaddition of tropone, aldehydes, and 2-aminomalonates.

**Scheme 25:**
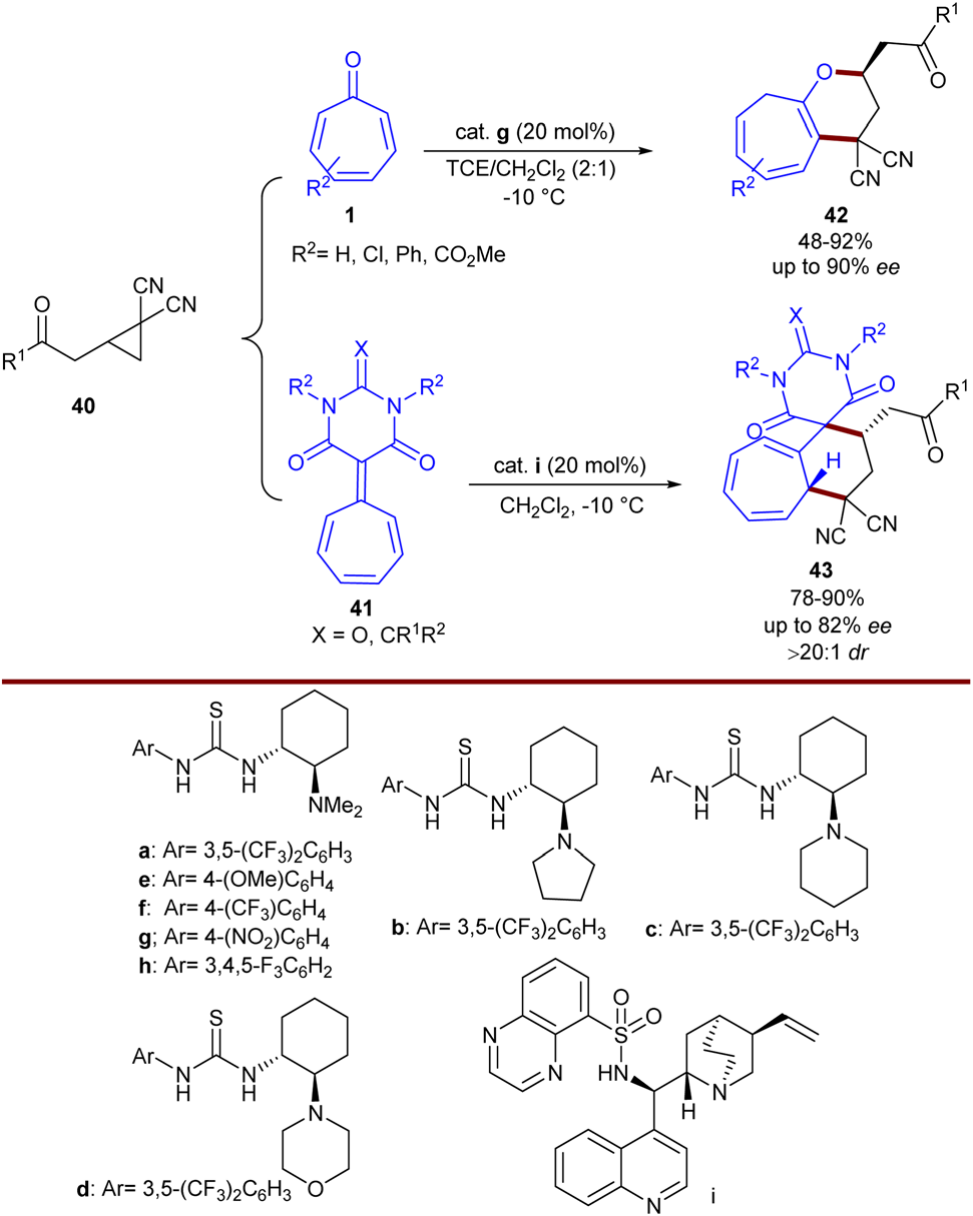
Brønsted base-catalyzed (8 + 3)-cycloaddition of donor–acceptor cyclopropanes with tropone.

Guanidine can act as an efficient organocatalyst in stereoselective (8 + 2)-cycloaddition of tropones 1 with azlactones 30 (Scheme 23).^49^ In this reaction, bifunctional guanidine acted as a hydrogen-bond-mediated catalyst to construct [5.3.0]bicyclic compounds 36 in up to 95% yield, >19 : 1 dr, and 96% ee. The bifunctional property of guanidine with an additional sulfonamide functional group (GS1–GS5) was found to be beneficial to the enantio-induction, while sterically hindered GS6 decreased the enantioselectivity. With the chiral catalyst GS8, the yield, enantioselectivity, and diastereoselectivity of the product significantly increased, making this catalyst the best one. When 2-substituted tropones were used, the cycloaddition occurred at the less substituted position. 2-Aryl tropones exhibited good compatibility with a little electronic effect (86–94% yield and 88–92% ee). For azlactones, 4-alkyl-substituted azlactones showed good compatibility regardless of the steric hindrance. The electronic feature of the 4-halo substituent on the phenyl ring of phenylalanine-derived azlactones had a slight influence on the enantioselectivity (92–94% ee) but significantly affected the reactivity (51–78% yield). According to the isolated [1,5]-H shift isomer, the X-ray crystal structure of a chiral guanidinium salt, and DFT calculations, the authors were able to explain the origin of enantio- and diastereoselectivity in the reaction. In addition, the synthetic utility of the method was demonstrated by the rapid transformation into enantioenriched α-amino acid derivatives and the gram-scale synthesis of the product (99% yield, 92% ee). Another use of NHC catalyst was demonstrated in Jørgensen's work (Scheme 24).^50^ A stereoselective high-order (6 + 4)-cycloaddition between tropone 1, aldehydes 34, and 2-aminomalonates 37 was performed to build a novel series of bridged azabicyclo[4.3.1]decane scaffolds 38 in the presence of a chiral phosphoric acid catalyst. The initial reaction of aldehydes and 2-aminomalonates resulted in the formation of azomethine ylides as key intermediates, followed by a 1,3-dipolar (6 + 4)-addition to tropone, providing the corresponding products in moderate to high yields, with excellent stereoselectivities (>95 : 5 dr and up to 99% ee). Bifunctional phosphoric acid catalyst can efficiently stabilize *in situ* generated azomethine ylides as a 1,3-dipole *via* hydrogen bonding interactions. The subsequent approach of tropone occurred from the less hindered position, as dictated by the bulky groups of the chiral catalyst. Jørgensen and his colleagues also reported chiral Brønsted base-catalyzed (8 + 3)-cycloaddition of donor–acceptor cyclopropanes 40 with tropone 1 (Scheme 25).^51^ Screening of various chiral organocatalysts showed that the organocatalyst g is the best choice. DFT calculations demonstrated the important role of the optically active bifunctional Brønsted base g in the activation of both the donor–acceptor cyclopropane and tropone through hydrogen-bonding interactions, leading to a stepwise manner with diastereo- and enantioselective ring-closure. The desired products 42 were constructed in moderate to high yields (21–92%) with excellent enantioselectivities (up to 92% ee). Besides, tropone derivative 41 can also be incorporated in the cycloaddition with cyclopropane 40 in the presence of another chiral organocatalyst I, yielding polycyclic compound 43 in good to high yields with high enantioselectivities.

#### 2.2.2. Base-catalyzed cycloaddition reactions involving tropone

**Scheme 26:**
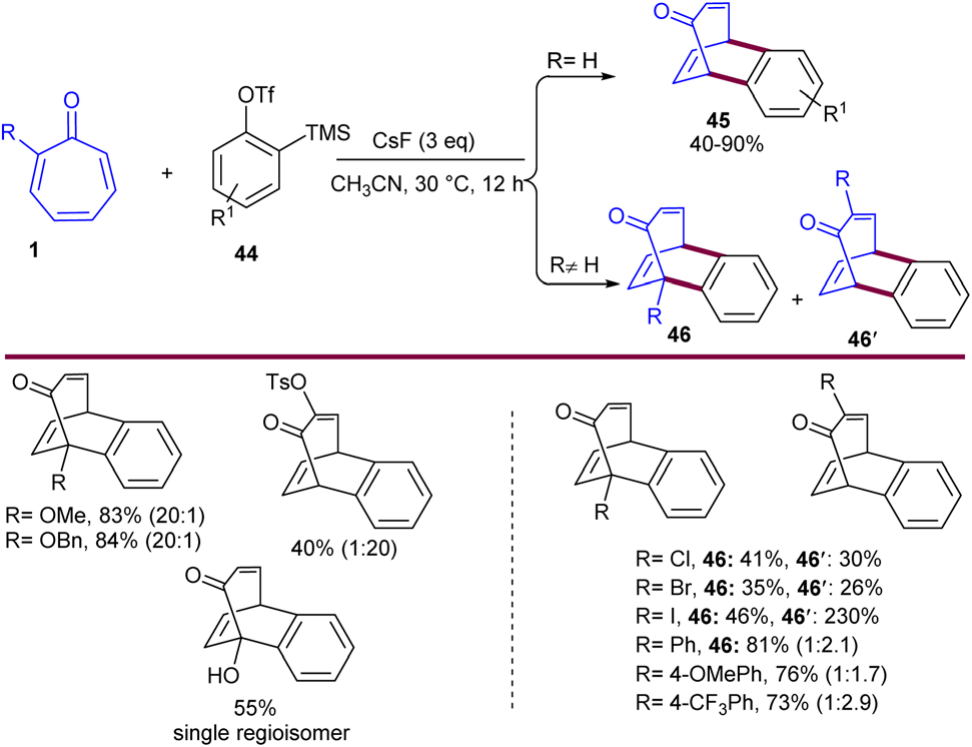
Diels–Alder reaction of tropones with 2-(trimethylsilyl)aryl triflates.

Diels–Alder reaction of tropones 1 with 2-(trimethylsilyl)aryl triflates 44 could be occurred in the presence of CsF as a base (Scheme 26).^52^ The base promoted the formation of a benzyne intermediate, which underwent (4 + 2)-cycloaddition with tropone to obtain benzobicyclo[3.2.2]nonatrienone derivatives 45. Variation of tropones, including electron-donating groups (OMe, OTs, OBn), exhibited 20 : 1 regioselectivity, whereas halogen (Cl, I, Br) and aryl groups resulted in moderate regioselectivity. This reaction was also applicable for tropolone, producing cycloadduct 46 as a single regioisomer in moderate yield (55%). The observation of this single regioisomer indicated that the Diels–Alder reaction proceeds faster than the aryne O–H insertion. The photophysical properties of some of the final products were investigated, which showed weak fluorescence absorption/emission at 270–290 nm.

**Scheme 27:**
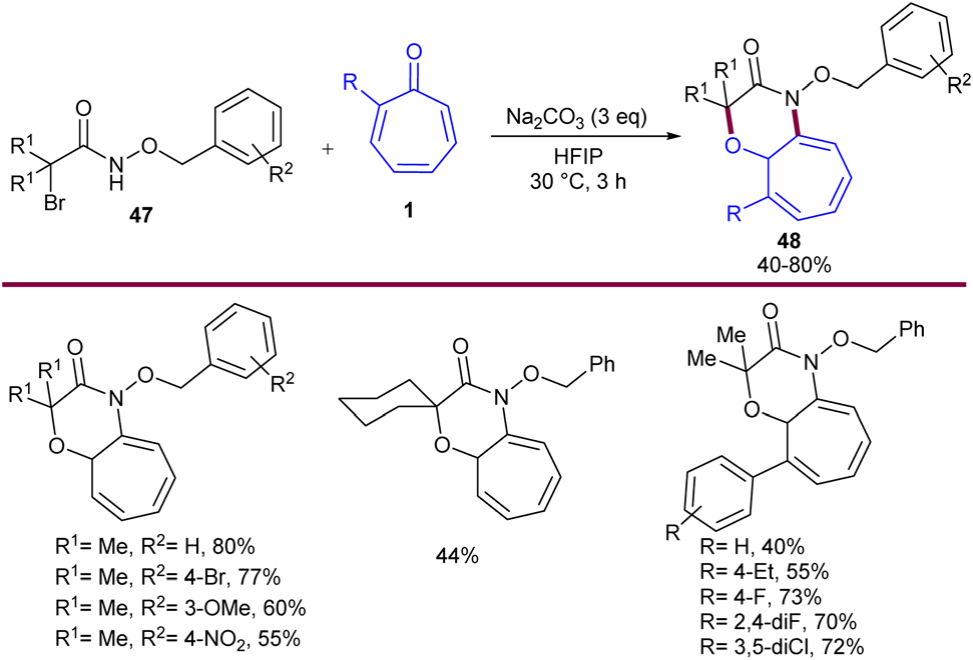
Na_2_CO_3_-promoted (8 + 3)-cycloaddition of tropones with azaoxyallyl cations.

**Scheme 28:**
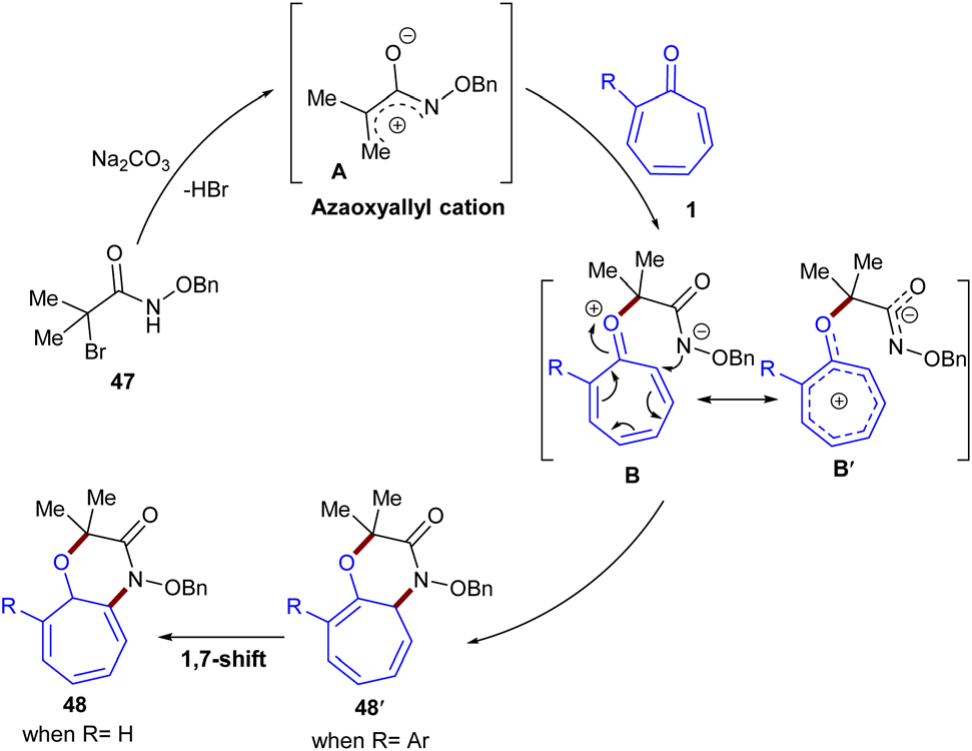
Plausible mechanism for Na_2_CO_3_-promoted (8 + 3)-cycloaddition of tropones with azaoxyallyl cations.

**Scheme 29:**
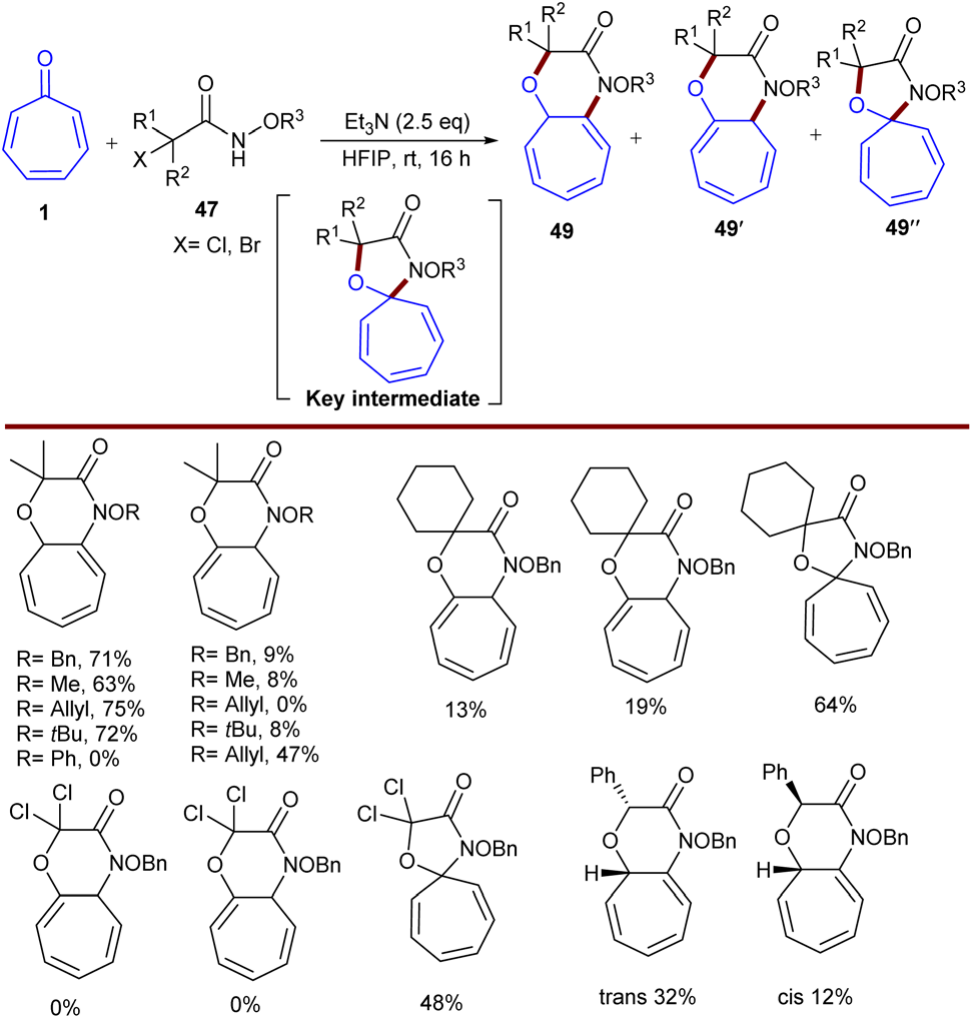
Et_3_N-promoted (8 + 3)-cycloaddition of tropones with azaoxyallyl cations.

In 2019, Roy *et al.* established base-mediated (8 + 3)-cycloaddition of tropones 1 with azaoxyallyl cations 47 (Scheme 27).^53^ Mechanistically, the reaction proceeded through the formation of the azaoxyallyl cation A from the interaction of the base with α-bromo hydroxamate 1. The nucleophilic addition of 47 to A led to the zwitterionic intermediate B, which could be in equilibrium with the stable tropylium cation B′. Then, B or B′ was subjected to intramolecular cyclization to form (8 + 3)-adduct 48′ when 2-aryl tropone was used. However, for unsubstituted tropone, the initial (8 + 3)-adduct underwent a 1,7-H shift to obtain the desired product 48. It should be noted that the production of the more substituted olefin is a reason for the stability of the initially formed (8 + 3)-adduct for 2-aryl tropones (Scheme 28). To get information on the kinetics of this (8 + 3)-cycloaddition, the authors performed a competition reaction between tropone 1 and 4-chlorobenzaldehyde, in which (3 + 2)-cycloadduct derived from 4-chlorobenzaldehyde was formed in 42% yield, while (8 + 3)-adduct from tropone had only 3% yield. This result showed that 4-chlorobenzaldehyde can react 14 times faster than tropone in the reaction with azaoxyallyl cations. At the same time, similar (8 + 3)-cycloaddition of tropones 1 with azaoxyallyl cations 47 was reported by another group (Scheme 29).^54^ This reaction was carried out in the presence of Et_3_N as a base in HFPI as a solvent at room temperature. DFT computations demonstrated the origin of selectivity in the reaction and the formation of key spiro intermediate A that could undergo ring-expansion to access nitrogen-containing [7,6]-fused bicycles 49.

**Scheme 30:**
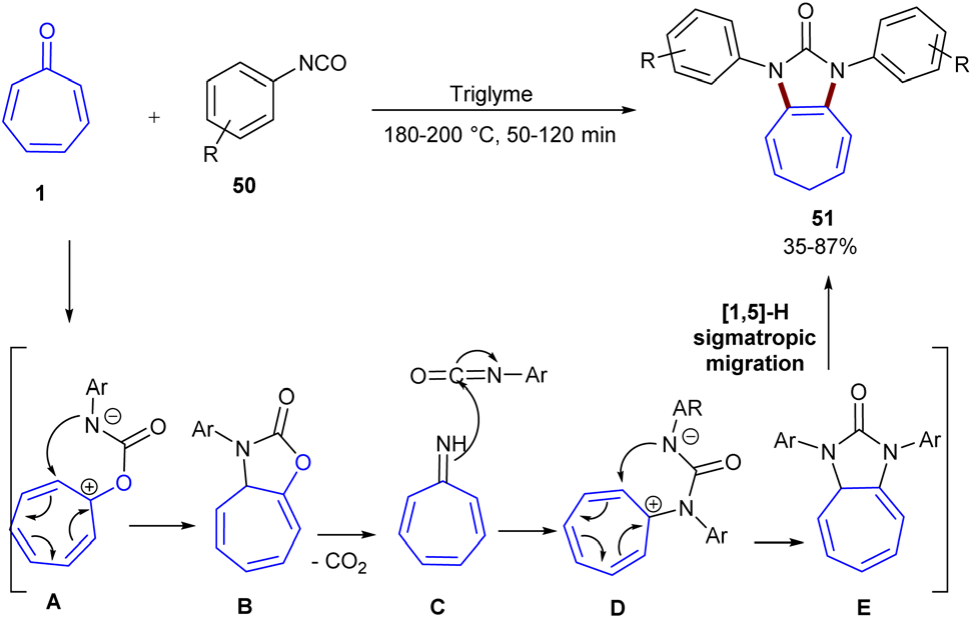
Catalyst- and oxidant-free reaction of isocyanates with tropones.

A high atom- and step-economical methodology for assembling troponoid derivatives incorporating imidazolin-2-one motifs 51 was reported by Hu and co-workers in 2020 (Scheme 30).^55^ In this regard, tropone reacted with isocyanates under catalyst- and oxidant-free conditions, using only triglyme as the solvent, at 180 °C for 50–120 min. The reaction was also applicable in other solvents, such as DMSO, DMI, tetraglyme, and bis(2-ethylhexyl)adipate, albeit with moderate yields of the product (28–73%). Generally, the cascade reaction involves the *O*-nucleophilic attack of tropone 1 to the carbonyl of isocyanate 50, followed by an intramolecular S_N_2′-like reaction. Tandem decarboxylation and *N*-nucleophilic attack on the second isocyanate, followed by sigmatropic [1,5]-hydrogen migration, afforded the desired product 51.

**Scheme 31:**
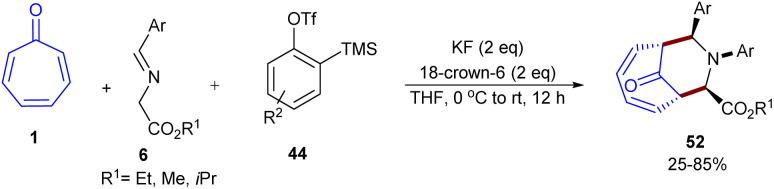
Metal-free (6 + 3)-cycloaddition of tropone, imino esters, and arynes.

**Scheme 32:**
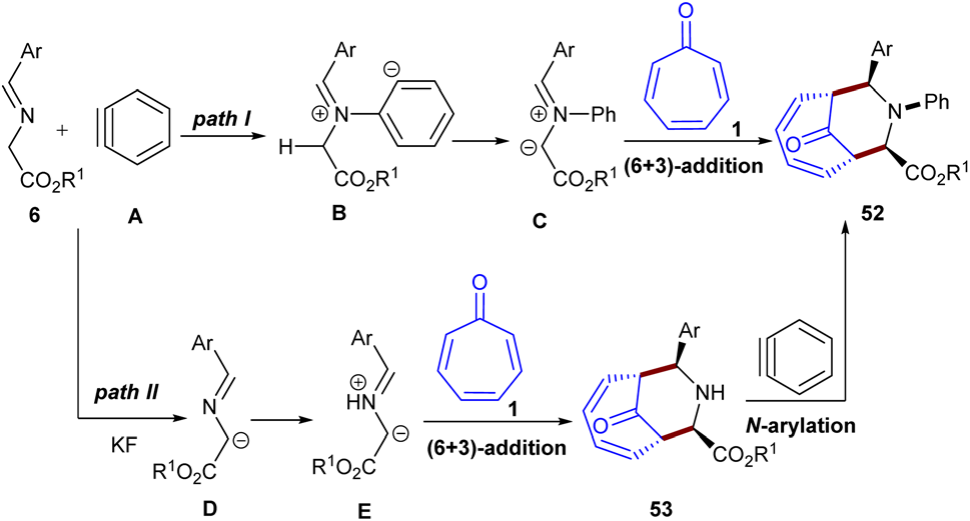
Mechanism metal-free (6 + 3)-cycloaddition of tropone, imino esters, and arynes.

Biju and co-workers developed a metal-free diastereoselective (6 + 3)-cycloaddition reaction between tropone 1, imino esters 6, and arynes 44 to make several bridged azabicyclo[4.3.1]decadiene compounds (Scheme 31).^56^ Two possible pathways were suggested for this transformation (Scheme 32). Path I involved the nucleophilic addition of imino ester 6 to aryne 44, generating the nitrogen ylide C*via* the zwitterion B, followed by diastereoselective (6 + 3)-cycloaddition with tropone 1, affording product 52. However, in path II, the presence of KF/18-crown-6 allowed the conversion of imino ester into *aza*-allyl anion E, which was protonated to form azomethine ylide F. Subsequent (6 + 3)-cycloaddition of F with tropone 1 delivered adduct 53, followed by *N*-arylation with aryne 44 to form product 52. To shed light on the mechanism, the authors performed the reaction in the absence of the aryne precursor, but did not observe the final product. The stop of the standard reaction after 2 hours also did not reveal compound 53, which ruled out pathway II.

#### 2.2.3. Catalyst-free cycloaddition reactions involving tropone

**Scheme 33:**
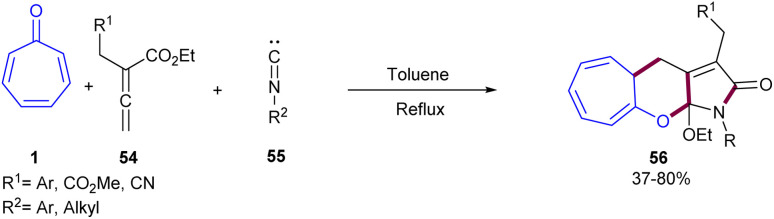
Multicomponent cascade cycloaddition involving tropone, allenoate, and isocyanide.

**Scheme 34:**
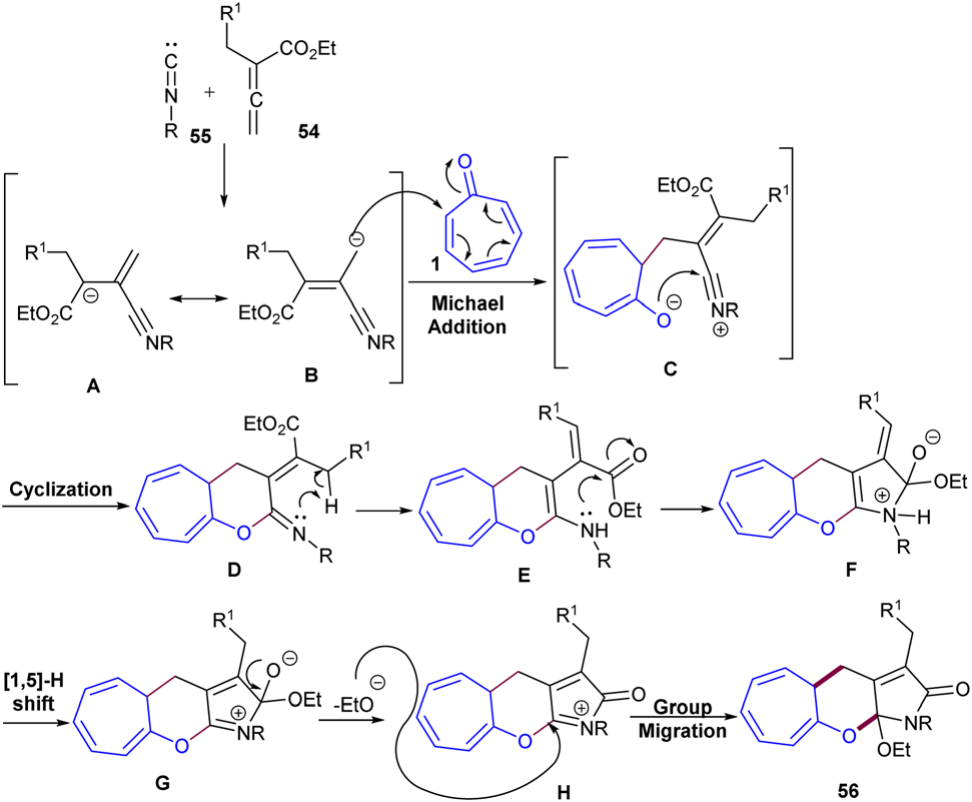
Proposed mechanism for multicomponent cascade cycloaddition involving tropone, allenoate, and isocyanide.

A rapid access to a 7,6,5-fused tricyclic framework was suggested by Jia and co-workers in 2014 (Scheme 33).^57^ For this purpose, tropone 1, allenoate 54, and isocyanide 55 were utilized as feedstock in toluene as a sole solvent under reflux conditions. The cycloaddition reaction was compatible with allenoate bearing electron-deficient and electron-rich aryl groups, as well as aryl and alkyl isocyanide. A plausible mechanism involving cascade (8 + 2 + 1)-cycloaddition, [1,5]-H shift, cyclization, and alkoxy group migration was proposed for the construction of highly unusual tricyclic products. Firstly, resonance-stabilized species A ↔ B was formed from the reaction of allenoate 54 and isocyanide 55. Then, the nucleophilic attack of these species on tropone 1 led to intermediate C, which underwent a [1,5]-H shift facilitated by an aryl or another electron-withdrawing group. The following cyclization and the second [1,5]-H shift afforded intermediate G. Finally, the elimination of the ethoxy group and nucleophilic addition furnished the desired product 56 (Scheme 34).

**Scheme 35:**
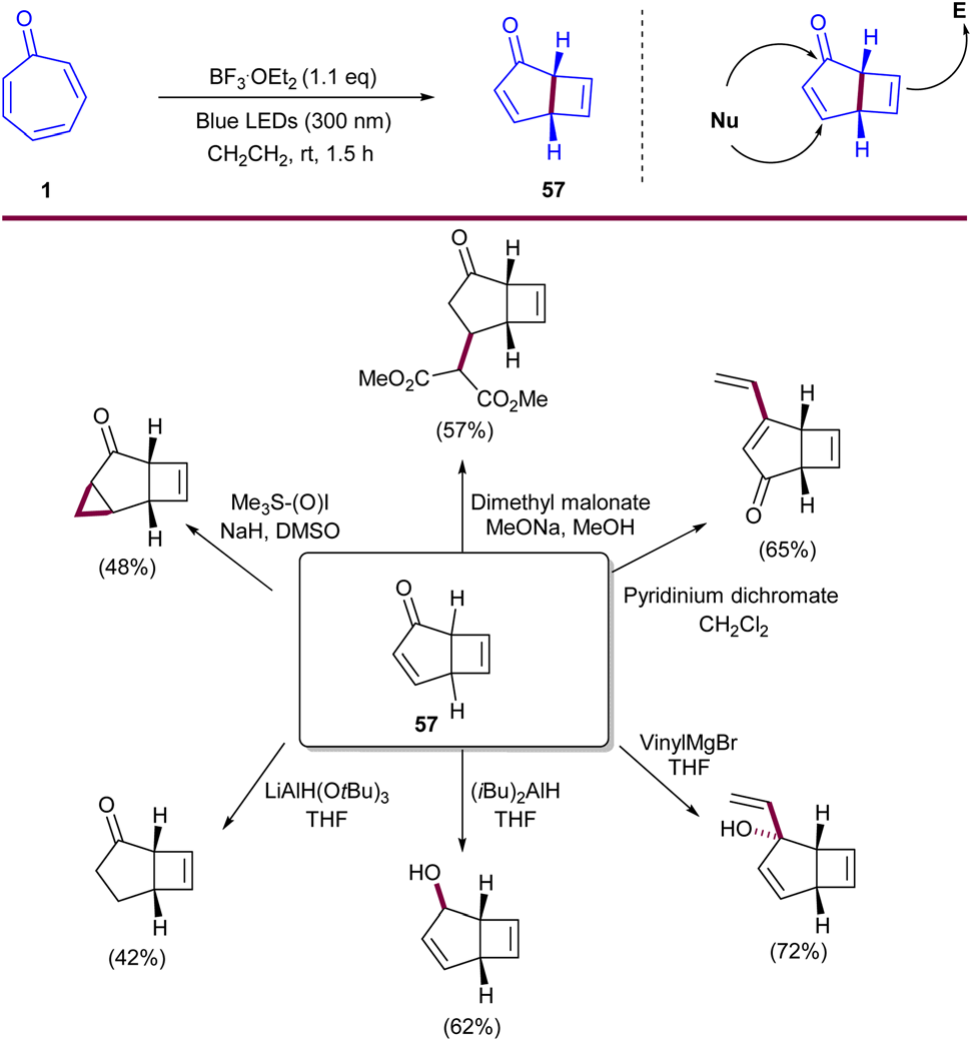
Lewis acid-catalyzed 4π-photocyclization of tropone.

**Scheme 36:**
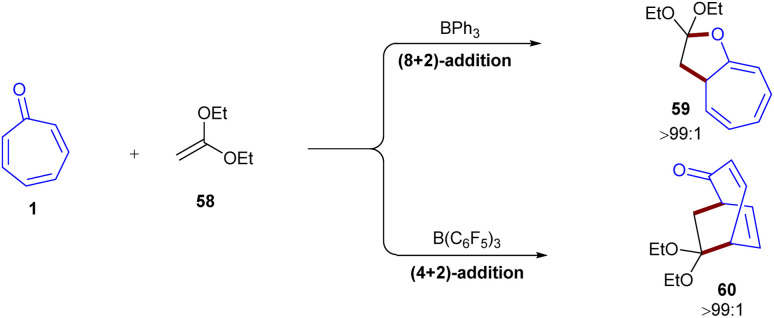
Lewis acids-catalyzed cycloadditions of tropone with ethene.

**Scheme 37:**
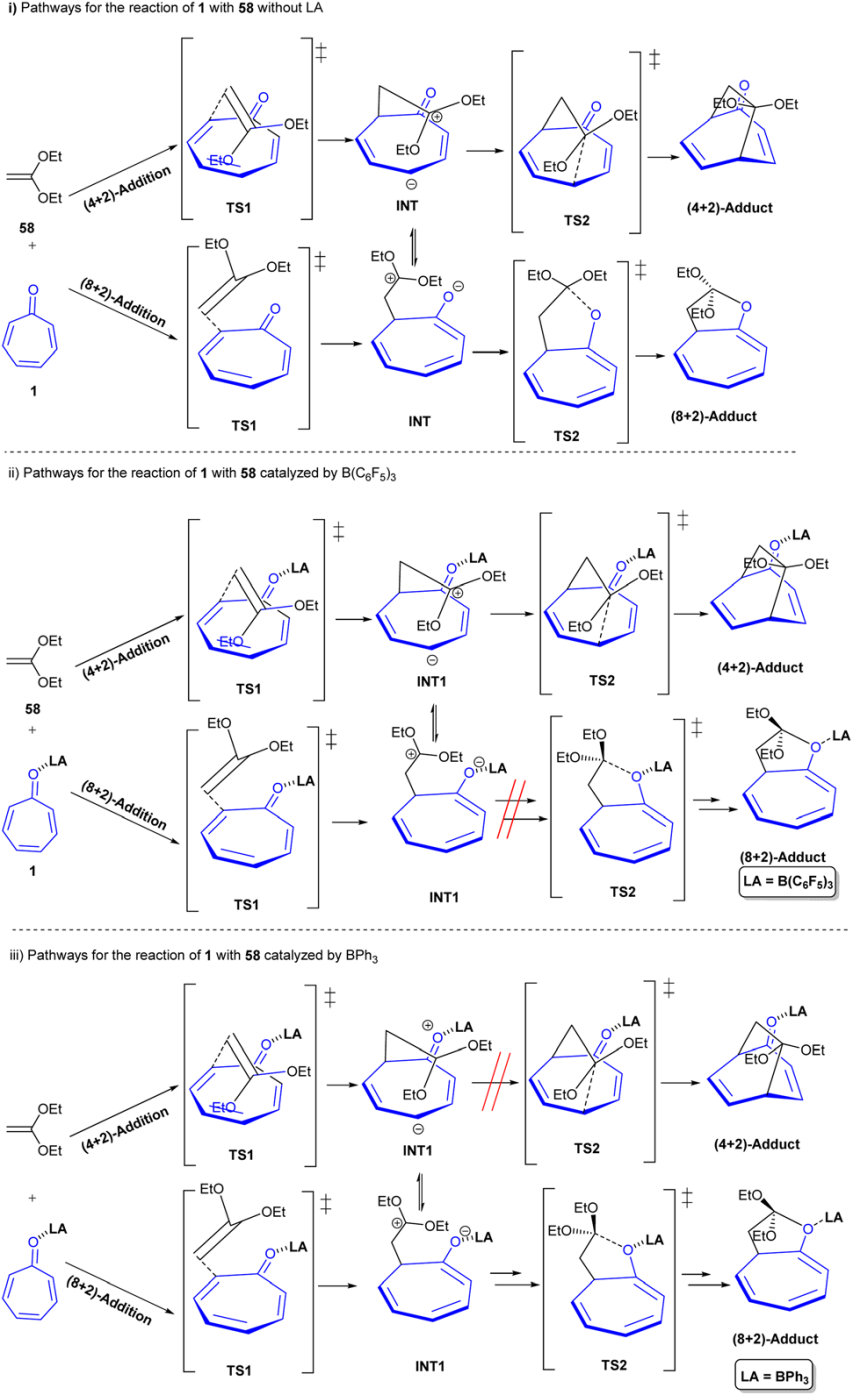
Lewis acids-catalyzed cycloadditions of tropone with ethene.

Lewis acid-catalyzed 4π-photocyclization of tropone was reported by Coote and co-workers (Scheme 35).^17^ BF_3_·OEt_2_ was used as a Lewis acid to promote 4π-photocyclization of tropone 1 under visible light irradiation to form bicyclo[3.2.0]-heptadienone 57. Notably, the lowest excited state of tropone complexed to a Lewis acid corresponded to a π–π* transition that enabled 4π-photocyclization. In contrast, in the absence of a Lewis acid, the lowest excited state of tropone corresponded to a prohibited n–π* transition from which 4π-photocyclization did not occur. Additionally, a variety of new rigid bicyclic scaffolds can be achieved through nucleophilic and electrophilic reactions of bicyclo[3.2.0]-heptadienone 57. Another use of Lewis acid catalyst in the cycloaddition of tropone 1 with 1,1-diethoxyethene 58 was observed (Scheme 36).^58^ The reaction was investigated in the absence of Lewis acid and in the presence of two kinds of Lewis acids, B(C_6_F_5_)_3_ and BPh_3_. In the absence of a Lewis acid, (8 + 2)- and (4 + 2)-cycloadducts 59 and 60 could be constructed through a stepwise reaction, wherein the C2 atom in tropone is bound with the C2 atom in ethene, and then the C5 atom in the former is bound with the C1 atom in the latter. For B(C_6_F_5_)_3_, firstly, the O atom in tropone is attached to the Lewis acid, and secondly, the C5 atom in tropone is attacked by the C1 atom in ethene. Although it was found that the attack of the O atom in tropone is less likely, favoring the (4 + 2)-cycloaddition in this case. In contrast, the attack of the O atom in the BPh_3_-attached tropone to the C1 atom in ethene was preferred over the attack of the C5 atom, proving the favorability of (8 + 2)-cycloaddition instead of (4 + 2)-cycloaddition. The attack of C5 or by O atom in tropone on the C1 atom in ethene both was controlled by the nucleophilicity of σ-lone pair electrons of the carbonyl O atom in the presence of Lewis acids (Scheme 37). Guerra and coworkers conducted a study on the 4-photocyclization mechanism of α-tropone derivatives.^59^ For this purpose, they chose tropone, 2-methoxytropone, and 2-cyanotropone as substrates and employed *ab initio* methods to investigate the 4π-photocyclization. Energy barrier calculations revealed that substituted α-tropones exhibit high barriers in the excited states, such as S_4_, S_3_, and S_2_. Although the electron-donating groups or the formation of hydroxytropenium ions can notably lower these barriers, thereby making 4π-photocyclization more feasible. It is worth noting that the 4-photocyclization of α-tropone derivatives primarily proceeds *via* low-lying excited states, with substituent effects influencing both reaction pathways and energy barriers. In addition, it was found that acid catalysts and the substitution of tropones with electron-donating groups reduce the energy barriers. Specifically, electron-donating groups on tropones promote degeneracy between S_1_ and S_0_ electronic states during the return to the ground state.

## 3. Functionalization reactions involving tropone

**Scheme 38:**
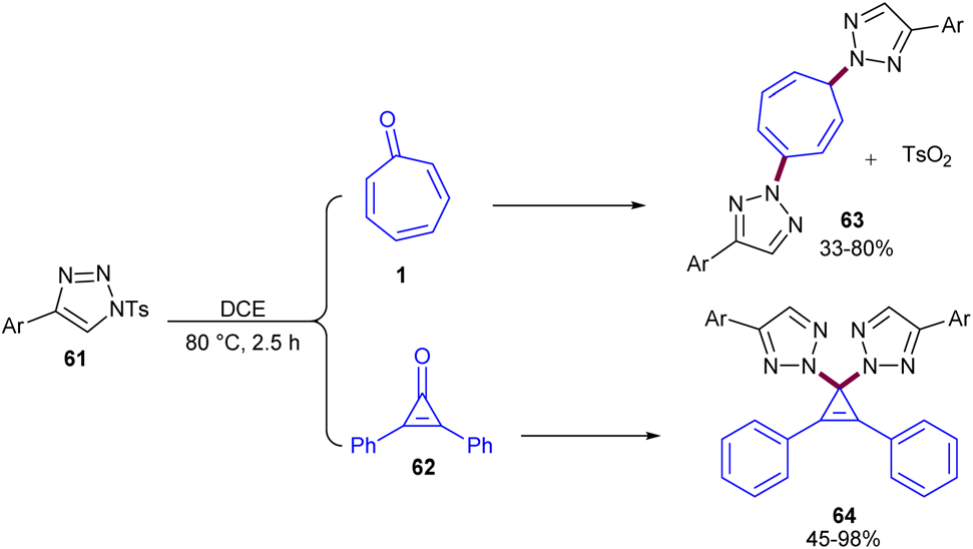
Ditriazolylation reaction of tropone with *N*^1^-sulfonyl-1,2,3-triazoles.

**Scheme 39:**
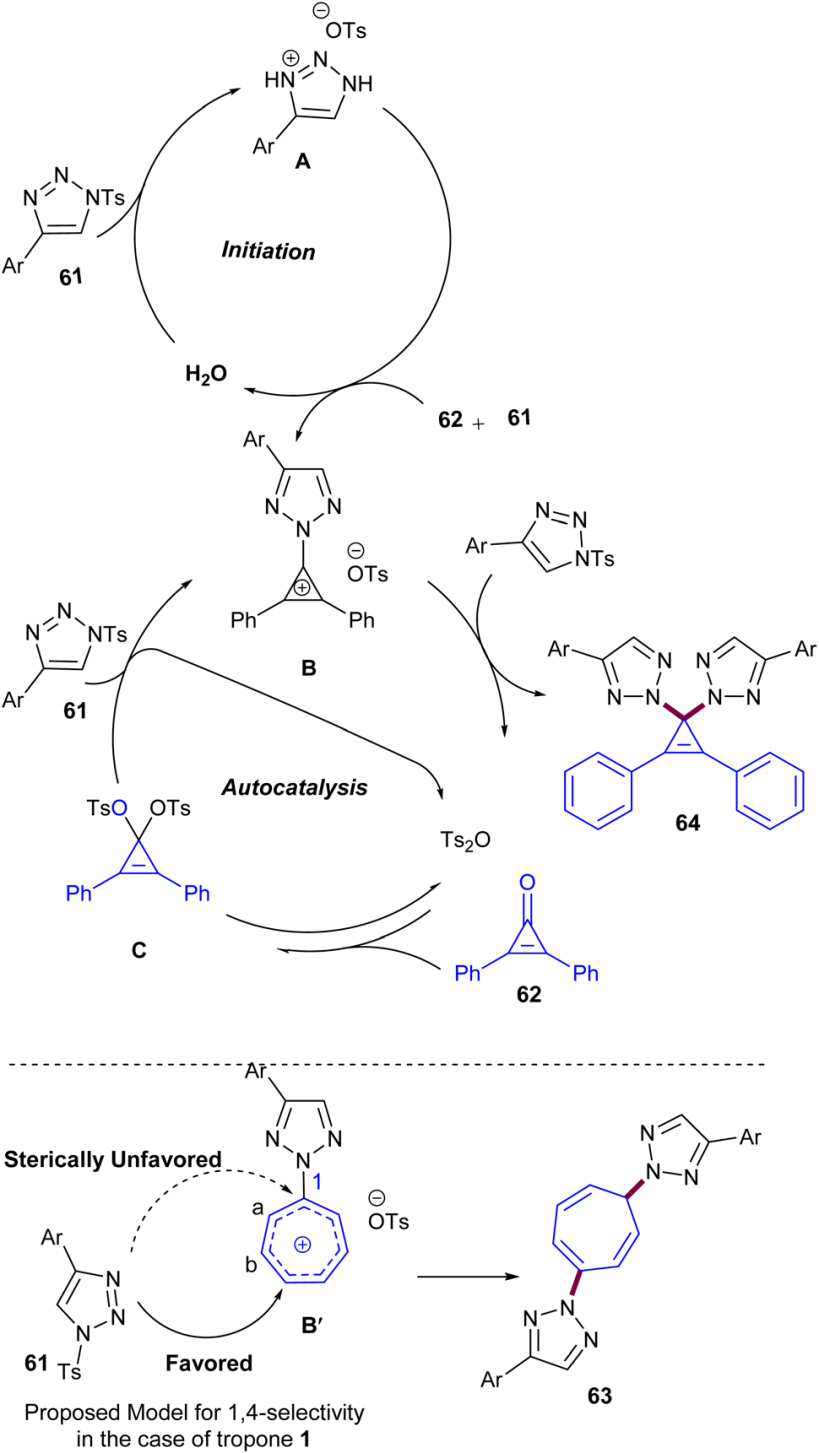
Proposed mechanism for ditriazolylation reaction of tropone with *N*^1^-sulfonyl-1,2,3-triazoles.

An unexemplified *N*^2^-selective autocatalytic ditriazolylation reaction between *N*^1^-sulfonyl-1,2,3-triazoles 61 with tropone 1 or cyclopropanones 62 was reported by Hao and co-workers in 2017 (Scheme 38).^60^ This protocol provides a simple and one-step transformation for the synthesis of *N*^2^-substituted bis(1,2,3-triazolyl) compounds 63 and 64. The reaction in the presence of a molecular sieve or at room temperature did not proceed, indicating the vital role of water as a solvent and the importance of heating in this reaction. Following mechanistic investigations, a proposed mechanism involved the hydrolysis of *N*^1^-sulfonyl-1,2,3-triazole 61 to generate intermediate A, which could act as an acid catalyst and catalyze the reaction of cyclopropanones 62 with another molecule of 61, providing intermediate B. Then, the remaining 61 acted as a nucleophile and reacted with B to form product 64 and Ts_2_O. Ts_2_O could formally be engaged in the autocatalytic cycle and react with 62 to generate intermediate C, which in turn reacts with two molecules of 61 consecutively to yield 64 through 1,4-disubstitution. It should be noted that due to the influence of steric hindrance in tropone, 1,4-disubstitution is more favored compared to 1,1-disubstitution in cyclopropanones. Meanwhile, Ts_2_O accumulated continuously until tropone 1 was consumed completely. A similar reaction mechanism was proposed for the reaction of tropone 1 and *N*^1^-sulfonyl-1,2,3-triazoles 61 (Scheme 39).

**Scheme 40:**
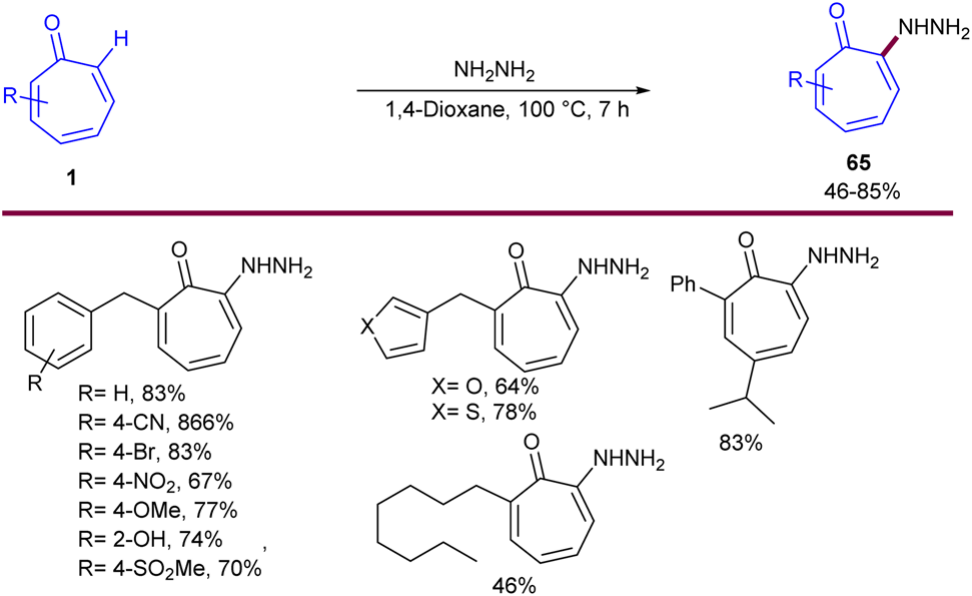
Metal-free C(sp^2^)–H bond amination of tropone with hydrazine.

**Scheme 41:**
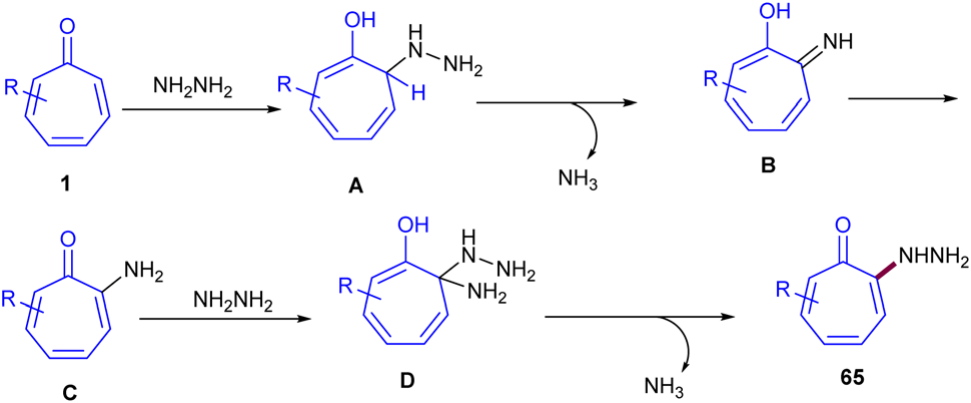
Proposed mechanism for metal-free C(sp^2^)–H bond amination of tropone with hydrazine.

**Scheme 42:**
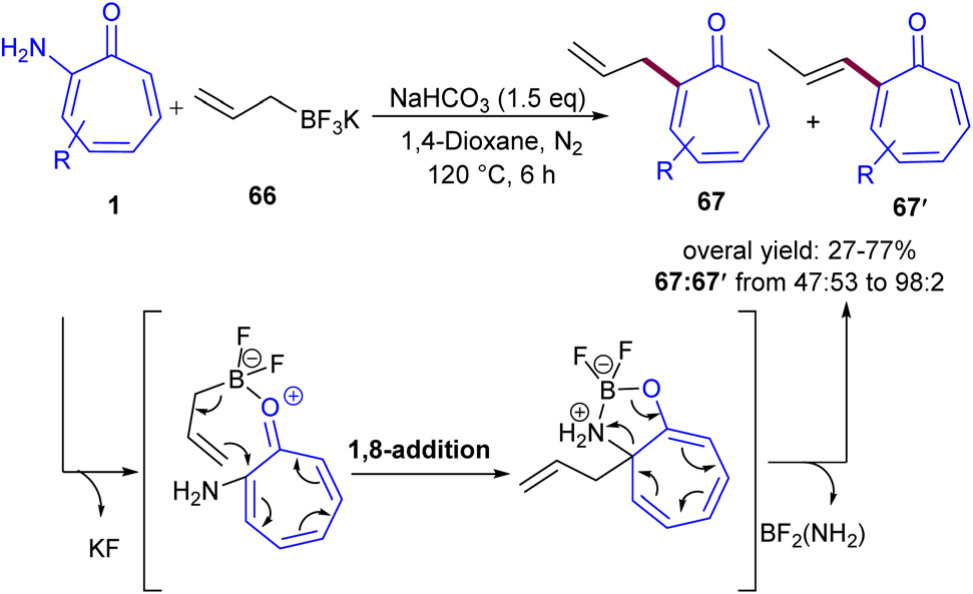
Metal-free C(sp^2^)–H bond allylation of tropone with potassium allyltrifluoroborates.

In 2024, Zhang and co-workers reported C(sp^2^)–H bond functionalization of the tropones 1 using hydrazine (Scheme 40).^61^ They introduced a metal-free and simple method for highly regioselective preparation of 2-hydrazinotropones 65*via* C–H amination process. By isolating 2-aminotropone intermediate A, the authors were able to propose a possible mechanism for this reaction, in which regioselective 1,8-addition of hydrazine to tropone 1 generated intermediate B, followed by the elimination of ammonia from to form imine C. Subsequent tautomerization afforded 2-aminotropone A, which underwent the second regioselective 1,8-addition of hydrazine and elimination of ammonia, producing the target product 65 (Scheme 41). The gram-scale reaction yielded the desired product in 841 mg, corresponding to a 62% yield. Further transformations of 2-hydrazinotropone to 2-chlorotropone, hydrazone-substituted tropone, or hydrazide-substituted tropone were also performed in this work. 2-Aminotropones 1 can undergo allylation reaction with potassium allyltrifluoroborates 66 (Scheme 42).^62^ The amino group was directly substituted with potassium allyltrifluoroborates 66 in the presence of a base, producing complex A. Then, the allyl group underwent an intramolecular 1,8-addition to tropone, generating intermediate B, which subsequently aromatized to the final product 67. The isomerization of 67 can result in the alkenylation product 67′ as a side product.

**Scheme 43:**
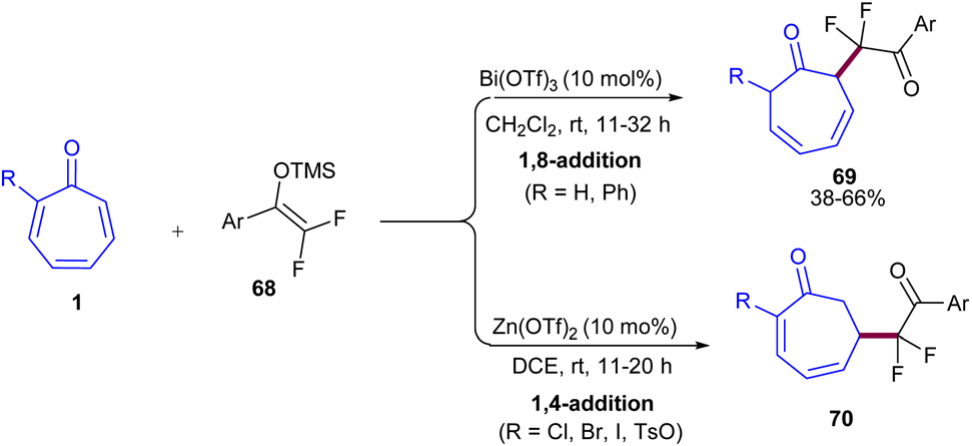
Bi(OTf)_3_-promoted reaction of tropone with difluoroenoxysilanes.

Functional groups at the C2-position of tropone can control regioselective conjugate addition (1,8- and 1,4-addition) of difluoroenoxysilanes to tropones (Scheme 43).^63^ In particular, 1,8-addition processed in the case of α-H, or α-Ph substituted-tropones 1, affording 3,5-cycloheptadien-1-ones bearing a *gem*-difluoroketone moiety 69. For tropones with α-halo, or α-TsO substituents, 1,4-addition adducts were formed as the major products. By studying DFT calculations, the authors found that 2-halo/TsO-substituted tropone has smaller HOMO–LUMO energy gaps relative to tropone, indicating that their electrons are more easily excited from the ground state to the excited state, so that they could undergo greater electron delocalization. And significant changes in their electron density upon excitation caused them to be favored for reaction sites far from the carbonyl of tropone, leading to 1,4-addition. In addition, the LUMO lobes on the C6 of 2-substituted tropones are greater than those of tropone, thereby increasing the tendency of C6 to participate in the 1,4-addition. At last, the synthetic utility of the method was demonstrated by the gram-scale synthesis of 1,8- and 1,4-addition products, yielding 1.20 g, 57%, and 1.42 g, 60%, respectively. Furthermore, the *in vitro* anti-proliferative activity of the obtained products against human colon cancer cells was investigated in this work.

**Scheme 44:**
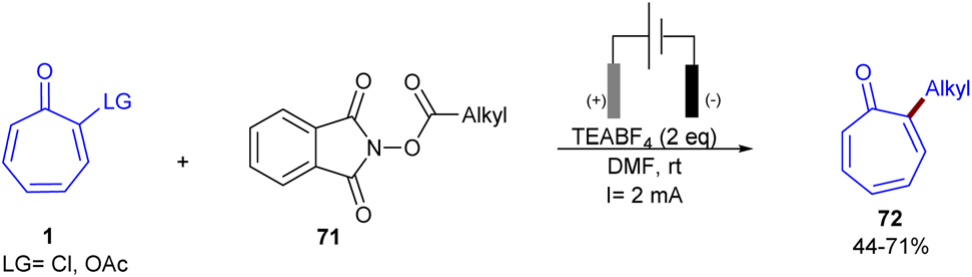
Electrochemical alkylation of tropones with *N*-hydroxyphthalimide esters.

**Scheme 45:**
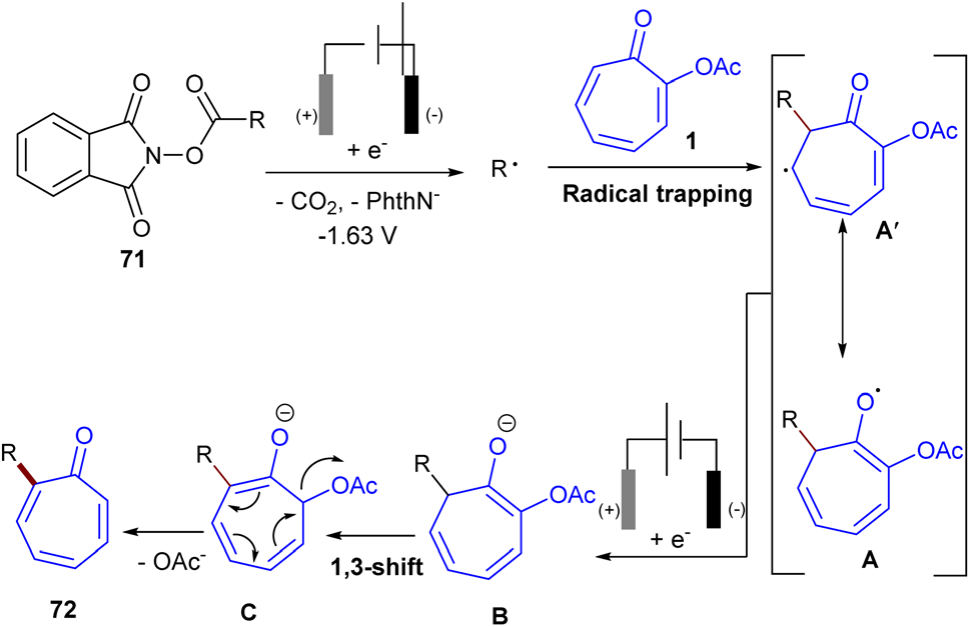
Possible mechanism for electrochemical alkylation of tropones with *N*-hydroxyphthalimide esters.

**Scheme 46:**
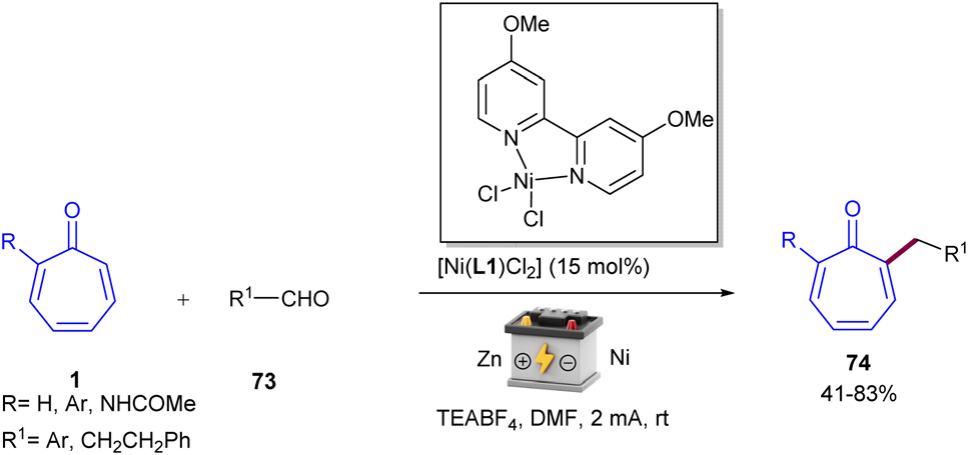
Ni-catalyzed electrochemical alkylation of tropones with aldehydes.

**Scheme 47:**
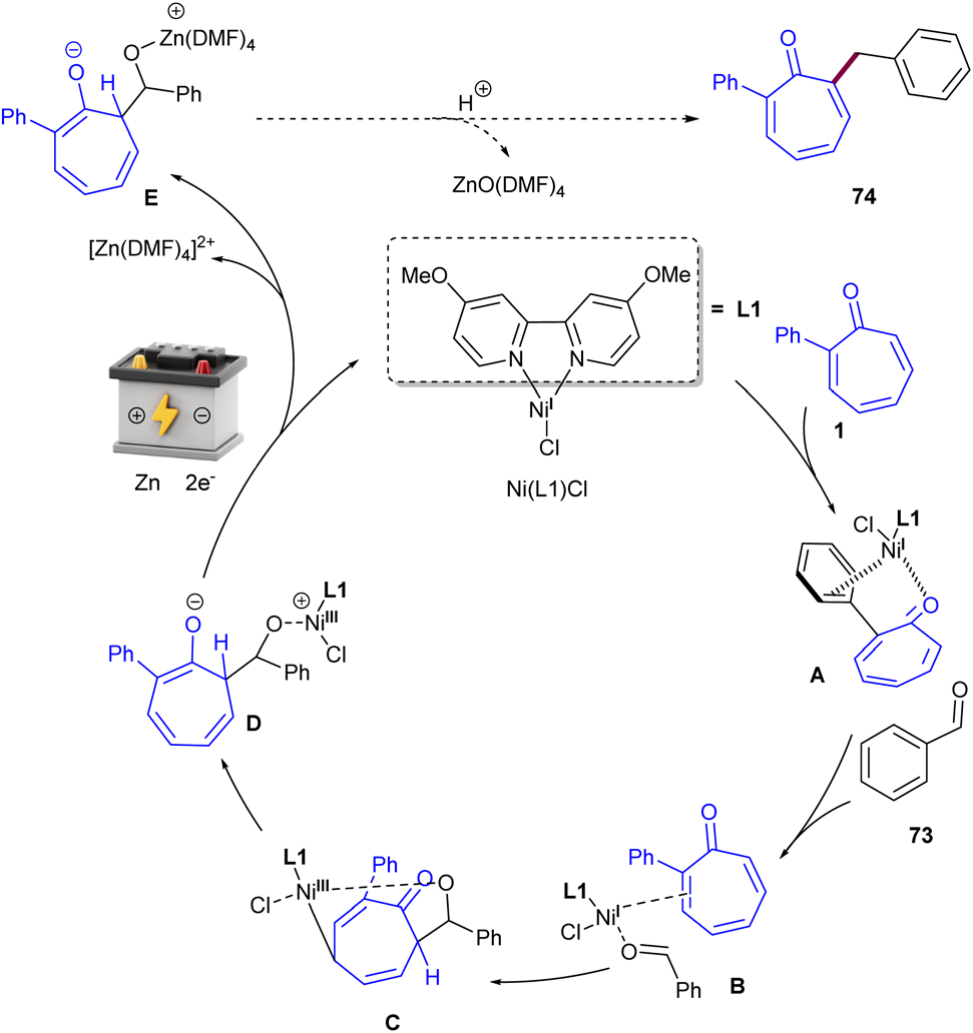
Catalytic cycle for Ni-catalyzed electrochemical alkylation of tropones with aldehydes.

Metal-free electrochemical functionalization of tropone 1 and substituted tropones with *N*-hydroxyphthalimide esters 71 as a redox-active ester was introduced by Bertuzzi's research lab (Scheme 44).^64^ This electro-induced alkylation reaction offers a highly regioselective method for synthesizing mono- and dialkylated tropones in moderate to high yields under very mild conditions. The steric hindrance of substituents in tropones had a significant impact on the site-selectivity, and alkylation occurred at the less hindered position. A possible mechanism was suggested for this electroreductive transformation, where a monoelectronic cathodic reduction of redox-active ester 71 resulted in the fragmentation of this molecule, delivering a nucleophilic radical species R˙. Then, R˙ was trapped chemoselectively at the α-position of tropone, providing a stable delocalized π-system intermediate A. Another cathodic reduction occurred to obtain the enolic form B, which underwent a [1,3]-H shift to form isomer C. The re-aromatization of C*via* elimination of the acetoxy group afforded product 72 (Scheme 45). Moreover, the late-stage functionalization of a bioactive compound (*i.e.*, colchicine analogue) emphasized the synthetic utility of this method. After a year, this research team introduced a nickel catalysis system for the alkylation of tropones under electrochemical conditions (Scheme 46).^65^ The reaction was found to proceed through the coordination of Ni with tropone 1 to complex A. Complex B forms after the Ni–O coordination with aldehyde 73, which results in a net transfer of electron-density from the Ni to the tropone. It should be noted that upon nickel coordination in complex B, the tropone moiety carried a negative charge, while the aldehyde was unperturbed. This interaction made the Umpolung of the reactivity of tropone and triggered the nucleophilic condensation to an aldehyde. The consequent C–C bond formation took place exclusively at the α-carbon of tropone, affording the organo-Ni(iii) complex C with a C(δ)–Ni bond. Then, the weak Ni–C bond broke, and a new Ni(iii)–O alkoxide D formed. The last electrochemical reduction of Ni(iii) to Ni(i) yielded the active species, and a Zn–Ni ion exchange between C and the Zn(ii) ions released from the sacrificial anode produced the Zn-alkoxide E through an exothermic step. Finally, E was converted to product 74 either directly or assisted by mild acidic media used during work-up (Scheme 47).

**Scheme 48:**
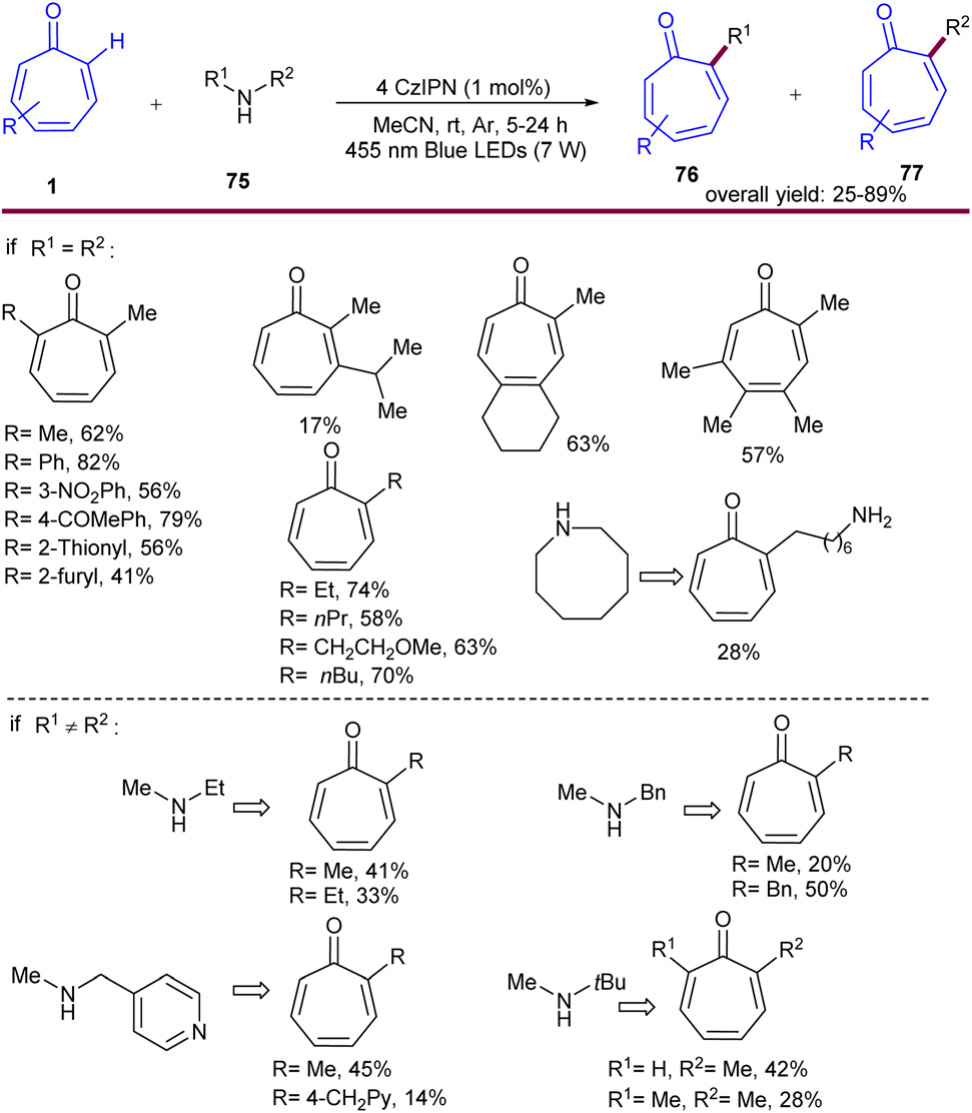
Photocatalytic alkylation of tropones with secondary aliphatic amines.

**Scheme 49:**
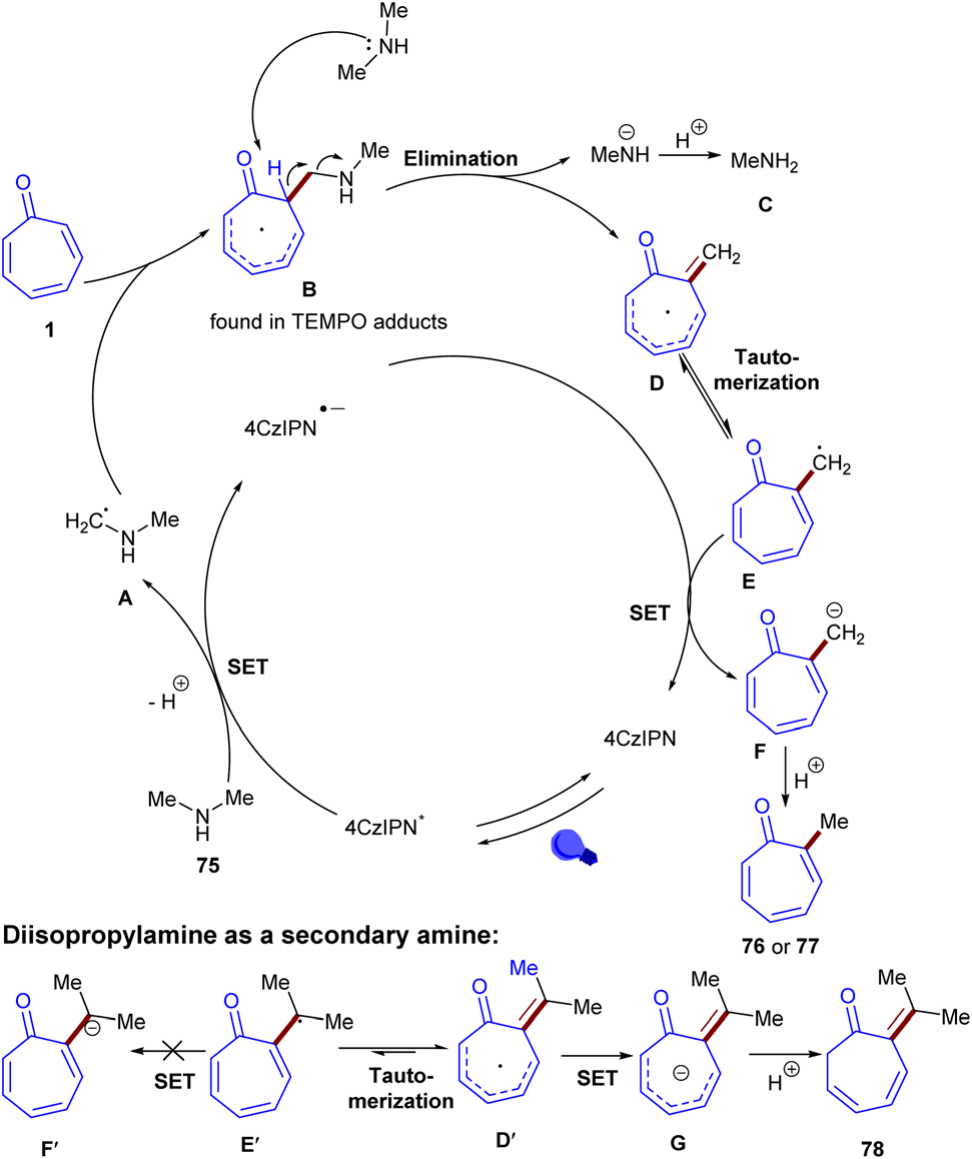
Proposed mechanism for photocatalytic alkylation of tropones with secondary aliphatic amines.

Another example of alkylation of tropone derivatives was catalyzed by 4CzIPN photocatalyst under visible light irradiation (Scheme 48).^66^ Screening of other photocatalysts, such as [Ru(bpz)_3_](PF_6_)_2_, thioxanthone, 4DPAIPN, or Eosin Y in this reaction was not successful. The reaction was initiated by photo-mediated excitation of 4CzIPN to the excited state 4CzIPN*, followed by attract of one electron from dimethylamine 75*via* a SET process, delivering a radical anion 4CzIPN˙^−^ and α-aminoalkyl radical A. The latter attacked tropone 1 to generate the amine-tropone radical B regioselectively. The acidity of the α-C(sp^3^)–H in the ketone can facilitate the following base-initiated elimination, catalyzed by dimethylamine 75, leading to the formation of the exocyclic double bond-containing radical D, accompanied by the liberation of methylamine C as a side product. In the next step, D was tautomerized to the methylene radical E, which subsequently attracted 1e^−^ from 4CzIPN˙^−^*via* a SET process. At the same time, the tropone-methyl anion F was generated and protonated to yield α-methyl tropone 1. It was realized that the presence of the exocyclic intermediate was substantiated by analysis of the mechanism leading to compound 78 with an exocyclic double bond. Since the inert tertiary carbon radical E′ was found to be unable to attract 1e^−^ from 4CzIPN˙^−^, thereby rendering the tautomerization from D′ to E′ unfavorable. As a result, the tropone radical D′ directly acquired 1e^−^ from 4CzIPN˙^−^ to obtain the tropone anion G, which protonated to yield 78. As the secondary amine functions dually as both an alkylation reagent and a base, thereby more than one equivalent of 75 should be suffice (Scheme 49). This protocol was also amenable to late-stage functionalization of some bioactive molecules and the transformation of fortunolide A into cephafortunoids A and B.

## 4. Conclusions

Over the last decade, attention to tropone synthetic chemistry has grown rapidly, resulting in the publication of elegant total syntheses of tropone-based bioactive molecules. As shown in this review, tropone has served as a 2π component in (2 + 4)-cycloadditions, a 4π component in (4 + 2)- and (4 + 6)-cycloadditions, a 6π component in (6 + 3)- and (6 + 4)-cycloadditions, and an 8π component in (8 + 2)-cycloadditions.

However, the electron-deficient nature of tropones limits Diels–Alder (4 + 2)-cycloadditions. One method for overcoming the low reactivity of tropones is to increase their nucleophilicity, enabling them to be used as dienes in normal electron demand Diels–Alder reactions.

For higher-order cycloadditions of tropones, one challenge is that the reaction often requires harsh conditions (high pressure or temperature) or yields are low, as the reaction requires overcoming the electron-poor nature and (4*n* + 2) π-aromatic character of the seven-membered ring. Another limitation in conjugate additions involving tropones is regio- and stereoselectivity, likely due to the challenges in controlling selectivity. Therefore, the development of site- and stereoselective conjugate additions of tropones that are capable of incorporating another pharmacophore to tropone is highly desirable, and important in medicinal chemistry.

For instance, the use of chiral ligands in metal-catalyzed reactions, or chiral organocatalysts, especially NHC catalysts, can provide an efficient and practical, highly stereoselective reaction systems that are desirable, and important in medicinal discovery.

We hope that this review article will help researchers explore the hidden aspects of tropone's nature and reactivity, thereby utilizing this valuable building block in organic transformations.

## Conflicts of interest

There are no conflicts to declare.

## Data Availability

No primary research results, software or code have been included and no new data were generated or analysed as part of this review.
